# Computational analysis and experimental verification of donor–acceptor behaviour of berberine, and its co-oligomers and co-polymers with ethylenedıoxythıophene

**DOI:** 10.1038/s41598-023-47541-7

**Published:** 2023-11-18

**Authors:** R. M. Gamini Rajapakse, Benjamin R. Horrocks, H. M. N. P. Gunarathna, A. U. Malikaramage, M. G. S. A. M. E. W. D. D. K. Egodawele, W. H. M. R. N. K. Herath, Lahiru Sandakelum, V. M. Y. S. U. Bandara, W. V. N. S. Bowatta, J. M. Susanthi Jayasinghe, V. N. Seneviratne, Udayana Ranatunga, L. L. K. Perera, S. M. Dassanayake, Chandana P. Udawatte

**Affiliations:** 1 Department of Chemistry, University of Peradeniya, Peradeniya, 20400 Sri Lanka; 2 School of Natural and Environmental Sciences, Newcastle University, Newcastle Upon Tyne, NE1 7RU UK; 3 Department of Decision Sciences, University of Moratuwa, Katubedda, Moratuwa, Sri Lanka; 4 Department of Physical Science and Technology, Faculty of Applied Sciences, Sabaragamuwa University of Sri Lanka, Belihuloya, Sri Lanka

**Keywords:** Electrochemistry, Theoretical chemistry, Conjugated polymers, Polymerization mechanisms, Natural product synthesis

## Abstract

The donor–acceptor (D-A) type of conjugated polymers has emerged as the paradigm of the third generation of electronically conducting polymers demonstrating improved infrared activity and intrinsic electronic conductivity. Judicious selection of donor (D) and acceptor (A) monomers for copolymerization can further fine-tune these properties. Notably, for such refinement, natural compounds provide many conjugated molecules with various functional groups. Berberine cation (Ber^+^) found in *Coscinium fenestratum* has extensive conjugation and contains both an electron deficient isoquinolium A moiety and electron-rich D-type methylenedioxy and methoxy groups. The incorporation of natural products in electronic materials is a novel area of research which opens a wide scope for future electronic and optoelectronic devices. Investigation of their fundamental properties via computer simulations is therefore important. In this study, quantum chemical calculations are performed using density functional theory (DFT) to investigate the electronic and optical properties of oligomers of Ber^+^ and 3,4-ethylenedioxythiophene (EDOT) and to explore the possibilities for homo-polymerization of Ber^+^ and its copolymerization with EDOT. It has been revealed that homo-polymerization is not favoured but copolymerization with EDOT is possible. As such, Ber^+^ was copolymerized with EDOT and the copolymers formed by electro-polymerization are extensively characterised and the D-A behaviour of the copolymers verified. Furthermore, the theoretical predictions have been compared with the experimental data.

## Introduction

The study of donor–acceptor (D-A) types of electronically conducting polymers has garnered significant attention in recent years due to their potential applications in optoelectronics, solar cells, and other electronic devices^1–7^. The D-A behaviour in polymers refers to the interactions between electron-rich and electron-poor units within a polymer. The electron-rich units, known as donors (Ds), may transfer electrons to the electron-poor units, known as acceptors (A)s^4^. This D-A behaviour plays a crucial role in determining the electronic and optical properties of the polymer in its intrinsic state without deliberate doping. Incorporation of alternating D and A units into a conjugated polymer allows control of the bandgap since the LUMO is mainly localised on the A unit and the HOMO on the D unit. Hence, judicious variation of D and A units controls the HOMO–LUMO separation, a key factor in the optical and redox properties of the materials.The electron-rich units typically consist of conjugated molecules, including homologous polyaromatic compounds and those containing heteroatoms such as nitrogen, sulphur, and oxygen. Conversely, electron-poor units are typically based on molecules with electron-withdrawing groups such as carbonyl, nitrile, quaternary ammonium, and cyano groups.

Berberine, a natural compound found in various plants, has been recognized as a potential donor–acceptor (D-A) material^8^. It contains a quaternary ammonium ion serving as the acceptor (A) moiety, with methylenedioxy and methoxy groups attached to phenyl rings acting as donor (D) moieties. Berberine exhibits several medicinally important properties, including antimicrobial, anti-inflammatory, and anti-cancer activities^9–11^. While the medicinal applications of berberine have been extensively studied, its utilization in the fabrication of electronically conducting polymers has not been explored thus far. Theoretically, Ber^+^ has the potential to undergo homo-polymerization, resulting in a poly(Ber^+^) homopolymer. However, oxidative polymerization may require a high positive potential due to the challenging oxidation of such a complex conjugated ion^12^. Alternatively, Ber^+^ can be copolymerized with easily oxidizable conjugated monomers such as EDOT or terthiophene (T_3_), following similar procedures employed in the synthesis of various complex D-A polymers^13–17^. Therefore, it is crucial to conduct theoretical investigations to explore the feasibility of copolymerization between Ber^+^ and EDOT.

**Figure 1:**
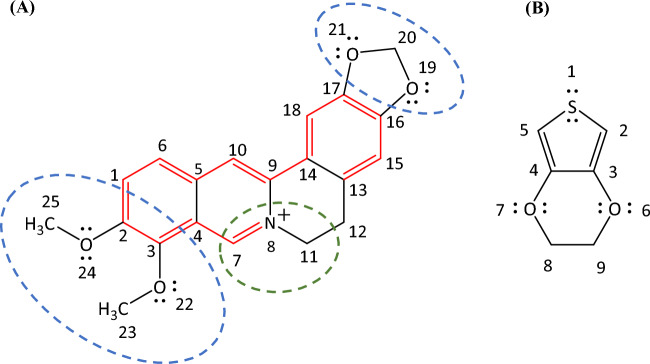
Chemical structures of (**A**) berberine cation (Ber^+^) showing electron rich (blue circled) and electron deficient (green circled) moieties and (**B**) ethylenedioxythiophene (EDOT) showing lone pairs of electrons capable of increasing the electron density of thiophene moiety.

However, to the best of our knowledge, there are no reports on either homo-polymerization of Ber^+^ or its copolymerization with easily oxidizable monomers such as EDOT. In the present context, it is crucial to investigate the donor–acceptor (D-A) behaviour of Ber^+^, along with its co-oligomers and co-polymers with EDOT. Figure 1A illustrates the D (highlighted in blue) and A (highlighted in green) segments, as well as the extended conjugation (highlighted in red) within the Ber^+^ cation. Figure 1B shows the electron-rich nature of EDOT.

The strength of D-A interactions can be influenced by several factors, including the polymer's chemical structure, degree of conjugation, nature of the interface between electron-rich and electron-poor units, and steric hindrance. The precise control of these interactions holds significant value in the design and advancement of polymers for diverse applications. Previously we have conducted investigations into the electrochemistry and optical spectroscopy of monomeric berberine cation with a view towards its eventual use in D-A conductive polymers^8^. In this work, we report DFT calculations on Ber^+^/EDOT copolymers and an experimental electrochemical investigation of the copolymerization and charge transport in a Ber^+^/EDOT copolymer.

## Experimental

### Computational approaches

Computer simulations were executed to analyse the electrochemical and spectroscopic attributes of Ber^+^ and EDOT, employing the Gaussian 09^18^ software. The optimized geometries of Ber^+^/EDOT were determined using DFT with the B3LYP functional and 6-31G(d) basis set. Excited state energies were estimated using TD-DFT at the same level of theory. We have previously ascertained that the B3LYP functional in conjunction with the implicit solvent model (default PCM in Gaussian 09) is sufficient to describe the optical and electrochemical behaviour of monomeric berberine cation^8^.

### Materials

Tetrabutylammonium hexafluorophosphate (TBAPF_6_), procured from Sigma Aldrich, and acetonitrile, obtained from Honeywell—Riedel-de-Haen, were used. The extraction and isolation of Ber^+^ from *Coscinium fenestratum* (tree turmeric) and its spectroscopic and electrochemical characterization were performed as previously reported^8^.

### Characterization

The UV–visible absorption spectra of the samples in acetonitrile and aqueous solution were recorded using a Shimadzu UV-1800 spectrometer (Serial No: A11635305394 CD). To perform scanning electron Microscopy (SEM) and energy dispersive X-ray analysis (EDX), the samples were deposited on fluoride-doped tin oxide (FTO) transparent conducting glass substrate surfaces. The samples were then cleaned with acetone to remove any residual solvent and air dried. The SEM images were obtained with a FEI Quanta 3D Dual Beam Microscope available in the high vacuum mode. The samples were analysed under analytical mode, with a working distance of 10 mm. The EDX and elemental maps were obtained with an X-MaxN 50 spectrometer (Oxford Instruments) mounted on the SEM machine.

### Electrochemistry

Cyclic voltametric studies of Ber^+^ and EDOT were conducted using a Metrohm PGSTAT204 electrochemical workstation (Serial No: AUT50184), following the methodology outlined in our previous work^8^. For each experiment, a solution was prepared by dissolving the respective monomer mixtures of Ber^+^ and EDOT in 25.0 mL of acetonitrile, resulting in concentrations of 5 mmol dm^–3^ in Ber^+^ and 10 mmol dm^–3^ in EDOT. To this solution, a tetrabutylammonium hexafluorophosphate (TBAPF_6_) background electrolyte (BGE) was added at a concentration of 0.10 mol dm^–3^. The electro-polymerization and cyclic voltametric (CV) measurements were performed using a one-compartment cell equipped with three electrodes. The working electrode (WE) was either a glassy carbon (GC) or an FTO, the reference electrode (RE) was a saturated calomel electrode (SCE), and a Pt-wire served as the counter electrode (CE). Before each experiment, the solution was degassed by purging with high purity nitrogen gas for 20 min, and a slow flow of nitrogen was maintained above the solution to prevent the re-entry of air. Electro-copolymerization was carried out by performing 25 repetitive CV cycles within the potential range of 0.0 V to + 1.8 V. The resulting polymer films on the WE surface were washed with acetone to remove any residual organic matter, and the CVs were recorded in the neat BGE. The CV data were recorded in the potential range of − 2.0 V to + 2.0 V to encompass all redox peaks of the materials, and a scan rate of 100 mV s^–1^ was employed, unless otherwise specified.

The Nyquist and Bode plots portraying alternating current (AC) impedance properties were generated over a frequency spectrum spanning 0.1 Hz to 1 MHz. These measurements were conducted at distinct direct current (DC) potential biases, selectively chosen, within the potential range of − 2.0 V to 2.0 V. This range comprehensively encompassed the pertinent information regarding the investigated electrochemical system.

## Results and discussion

### Infeasibility of homo-polymerization of berberine

**Figure 2:**
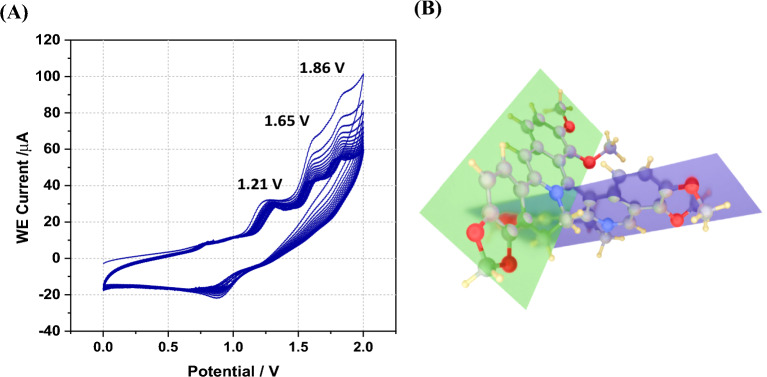
(**A**) Cyclic voltammetry of berberine in acetonitrile application of − 2.0 V to + 2.0 V with 100 mV s^–1^ scan rate. The working electrode was a 0.50 cm diameter glassy carbon disc, the reference was SCE and the concentration of berberine was 3.0 mM. The solution was purged with N_2_ to remove dissolved oxygen. The direction of change with scan number is to a decrease in the 1.65 V and 1.86 V peaks. (**B**) Ber^+^–Ber^+^ optimized using the B3LYP/6-31G(d) model chemistry. Planes of two Ber^+^ moieties in the Ber^+^-dimer are shown in purple and green.

Both DFT and TD-DFT calculations were performed to investigate the electronic structure of berberine dimers as an intermediate in the homopolymerisation of berberine. The structure of the dimer was optimised, and then vertical excitation energies were calculated to the lowest excited singlet (S_1_) and triplet (T_1_) states; the data is summarised in Table 1 alongside a comparison of the results for the monomeric berberine cation^8^. The HOMO and LUMO energies are lower in the dimer, owing to the electrostatic effect, but overall, the HOMO–LUMO gap increased by about 0.095 eV. Nevertheless, the energy of the S_1_ state is decreased in the dimer by 0.42 eV and the S_1_-S_0_ gap in the monomer has been shown to correlate well with the electrochemical gap^8^. The importance of this lowering lies in the mechanism of electropolymerisation, which for a typical conjugated monomer requires that the oligomers are oxidised at the same or a lower potential than the monomer^19,20^. However, this is insufficient on its own because it is necessary to consider the geometry of the dimer; the optimised geometry of the berberine dimer is shown in Fig. 2.

**Table 1:** The energies (reference to acetonitrile medium) of HOMO, E_H_, and LUMO, E_L_, of the berberine cation alone and interacting two cations which were calculated using B3LYP/6-31G(d) with implicit solvation by acetonitrile using the default PCM approach of Gaussian 09.

Sample	E_H_/eV	E_L_/eV	∆E(E_H_–E_L_)/eV	∆E (Excitation–Ground)/eV, [nm], (Excitation)^8^
Ber^+^	– 5.687	− 2.984	2.694	2.725, [455], (S_0_ → S_1_)
				2.189, [566], (S_0_ → T_1_)
Ber^+^–Ber^+^	− 5.902	− 3.113	2.789	2.304, [538], (S_0_ → S_1_)
				1.761, [704], (S_0_ → T_1_)

Excitation energies at the optimised geometry of the ground state were computed using TD-DFT. Monomeric berberine calculations from ref.^8^.

Our attempts to homopolymerize Ber^+^ in an acetonitrile medium, with TBAPF_6_ as the BGE, did not yield any deposition on the surface of the working electrode and the currents in consecutive CVs decreased. Figure 2A shows the CV of Ber^+^Cl^–^ in the potential range from – 2.0 to + 2.0 V.

**Figure 3:**
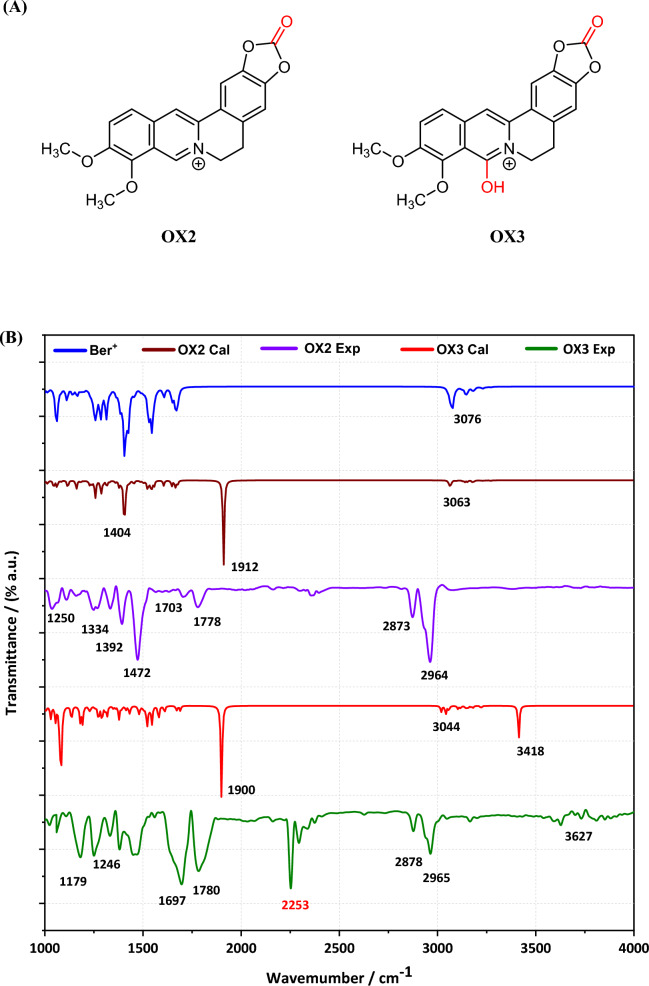
(**A**) Possible oxidized products (OX2 and OX3) of Ber^+^ in the potential range from 1.6 V to 2.0 V region. (**B**) Calculated IR spectra of Ber^+^ (blue), Oxidized product 2 (OX2 Cal–brown), Oxidized product 3 (OX3 Cal–red) and experimental FT-IR spectra of Oxidized product 2 (OX2 Exp–purple), Oxidized product 3 (OX3–green). IR frequencies of two oxidized products were calculated using acetonitrile solvent model and 6-31G(d) basis set. (a.u.–arbitrary units).

The CV of Ber^+^ in acetonitrile containing TBAPF_6_ given in Fig. 2A shows three distinctive oxidation peaks centred at + 1.21 V, + 1.65 V, and + 1.86 V and the latter two are due to the oxidation of Ber^+^ at high positive potentials resulting in compounds formed as shown in Fig. 3A. The first peak is characteristic of ${\mathrm{Cl}}^{-}$ to Cl_2_ oxidation as shown in our previous publication^8^. The repeated CVs did not yield any polymer deposited on the WE surface, and the current densities declined in the consecutive repeated scans. As such, it is clear that the oxidation of Ber^+^ does not result in electro-polymerization to yield a conductive poly(Ber^+^). The steric hindrance between two Ber^+^ cations prevent the normal coplanar arrangement of rings in conductive polymers as shown in Fig. 2B; we argue that this factor is partly responsible for the lack of success in achieving homopolymerisation of berberine. We must also consider whether the one-electron oxidation product of berberine undergoes other chemical reactions in competition with oligomerisation.

Based on FT-IR data, we uncover evidence supporting the structural resemblance between these compounds in our comparative analyses of calculated OX2^21^ and OX3 out of 12 structures and (Supplementary information SI Figs. 03, 04) and experimental OX2, OX3 oxidation products^12^. Notably, the FT-IR bands for several functional groups are nearly identical, indicating that they share a similar bonding nature. For the second oxidation product OX2, wavenumbers align for C–O stretching (–C–O–CH_3_) (OX2 Cal: 1181 cm^–1^, OX2 Exp: 1183 cm^–1^) ^22^, –C–N– stretching (OX2 Cal: 1359 cm^–1^, OX2 Exp: 1334 cm^–1^), –C–H bending (OX2 Cal: 1404 cm^–1^, OX2 Exp: 1392 cm^–1^)^23^, =C–H stretching (OX2 Exp: 1472 cm^–1^, OX2 Cal: 1486 cm^–1^)^24^, and –C–H_3_ stretching vibrations (OX2 Cal: 3063 cm^–1^, 3071 cm^–1^, and OX3: 3076 cm^–1^), further affirming structural similarity with an additional band centred at 1778 cm^–1^ indicating the presence of a carbonyl group at some position in the oxidised product OX 2 The most likely position for oxidation to form a carbinyl group is the methylenedioxy carbon atom that has also been suggested by the Referee 1 also. We, therefore, propose the structure OX2 given in Fig. 3A as the most likely oxidation product appearing at + 1.65 V wrt Ag/AgCl/KCL(sat.) electrode. This may have been formed by the reaction with traces of oxygen present in the solution although it was purged with nitrogen gas for about 30 min. This is further evidenced by the observation of the increased peak current at + 1.65 V when the solution is purged with air. For the third product, OX3, an additional band centred at 3627 cm^-1^ in the experimental spectrum is due to –OH group stretching at which appears at 3416 cm^-1^ for the OX3 in the calculated spectrum^22,25^, OX 3 could be formed when OX2 is reacted with traces of water present in the solution. Therefore, the possible structure for the oxidation product appearing at + 1.68 V wrt Ag/AgCl/KCl(sat.) is the OX 3 in Fig. 3A.

### Simulation studies of the copolymerization of berberine (Ber^+^) with ethylene dioxythiophene (EDOT)

We calculated the HOMO and LUMO energy levels of Ber^+^ and EDOT in three distinct media: vacuum, water, and acetonitrile. The primary aim of the theoretical study was to determine the solvent medium that yields the smallest HOMO–LUMO energy difference for Ber^+^ and EDOT in combination. The obtained results serve to provide valuable insights into the electronic properties of Ber^+^ and EDOT within different solvents, aiding in the comprehension of its behaviour across various media. The outcomes of these calculations are presented in Table 2.

**Table 2:** The energies of the HOMO (E_H_), and LUMO (E_L_) with reference to vacuum, water and acetonitrile of Ber^+^ cation and EDOT in selected media calculated using the B3LYP functional and 6-31G(d) basis set together with respective energy gaps, E_g_.

Solvation model	Ber^+^			EDOT		
	E_H_/eV	E_L_/eV	E_g_/eV	E_H_ /eV	E_L_/eV	E_g_/eV
Vacuum (g)	− 8.495	− 5.549	2.846	− 5.745	− 0.033	5.712
Water	− 5.858	− 2.613	3.245	− 5.914	− 0.156	5.758
Acetonitrile	− 5.687	− 2.984	2.694	− 5.909	− 0.141	5.768

**Figure 4:**
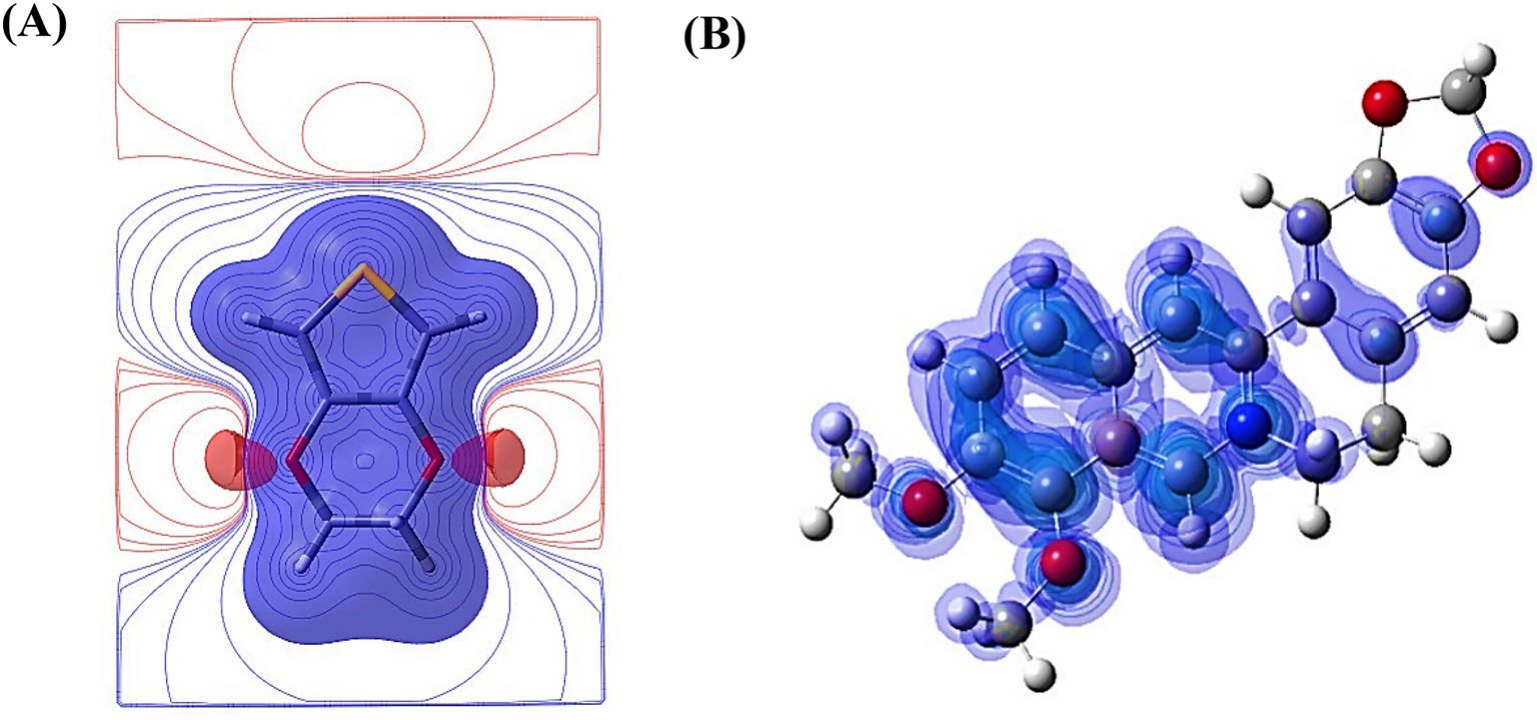
(**A**) Distribution of electrostatic potential map (ESP) map on the EDOT (positive contours in blue and negative contours in red). (**B**) Spin density diagram of berberine (Ber^2+^) cation.

The data in Table 2 indicate that although the HOMO energies in Ber^+^ and EDOT are similar in polar media, the LUMO energy of Ber^+^ is much lower by about 2.5 eV than that of EDOT. The calculated electrostatic potential (ESP) for the ground state of EDOT was calculated at the B3LYP/6-31G(d) level. Figure 4A depicts the analysis of high electron density contours, which show that carbon atoms numbered 2 and 5 exhibit a greater inclination to donate electrons to A molecules like in Fig. 4B. This can be attributed to the presence of two lone pairs on the S atom that are bonded to these carbon atoms.

**Figure 5:**
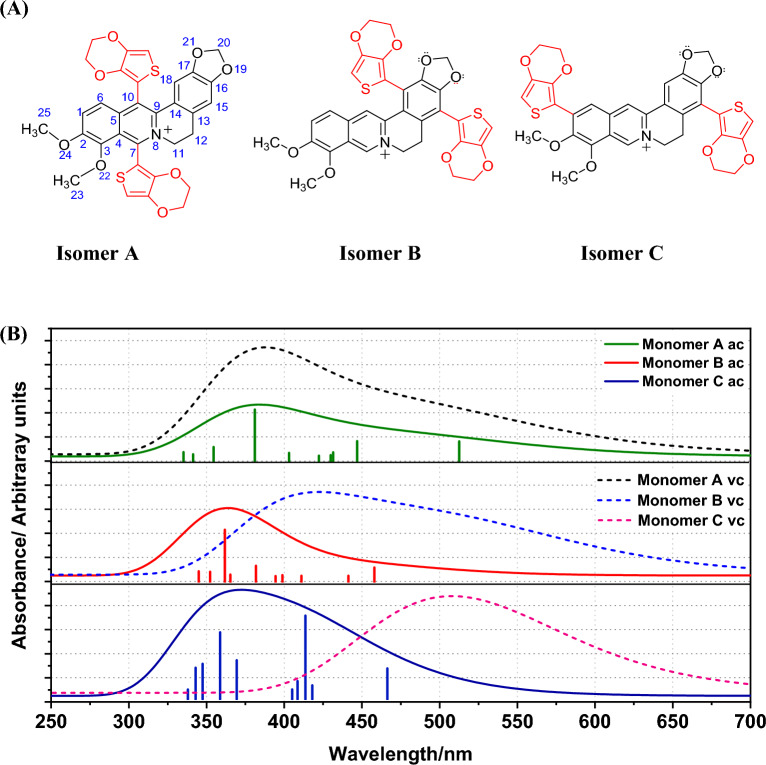
(**A**) The proposed monomer structures of EDOT-(Ber^+^)-EDOT when Ber^+^ and EDOT reacted in 1:2 molar ratio. (**B**) Simulated absorption spectra of three possible (EDOT-Ber^+^-EDOT) monomers. Model chemistry B3LYP/6-31G(d) was used with vacuum (dash line) and acetonitrile (solid line) solvent models.

In our previous studies, we found that if 1:1 stoichiometry of the two monomers A and B is used, the electro-co-polymerization leads to the formation of an alternating copolymer, (A-B)_n_. Additionally, if x:y stoichiometric ratio is used the resulting polymer is a copolymer [A_x_-B_y_]_n_^4,14,26,27^. However, it is important to carefully control reaction conditions and other relevant parameters to understand the behaviour of the cations and the reaction mechanism. It is well established that electro-polymerization of EDOT leading to PEDOT undergoes via 2 and 5 positions (two ortho positions to S in the thiophene unit). However, several possible regioisomers of Ber^+^-EDOT co-oligomers are possible (Fig. 5A). Below we use A, B and C to denote the pattern of coupling of Ber^+^ and EDOT irrespective of the EDOT/Ber^+^ ratio *n*.

To find out energetically the most favourable regioisomers, DFT calculations were performed to investigate the interaction between Ber^+^ and EDOT and the data are summarised in Tables 3 and 4. The removal of a hydrogen atom from carbon number 5 in isomer A is impeded by the presence of the electron-rich oxygen atom and electron cloud in the O-CH_3_ group results in steric hindrance, which complicates the combination of the electron rich EDOT molecule with carbon 5. The simulation data shows that the isomer C has the lowest overall electronic energy, with B lying 0.19 eV higher and isomer A at 0.70 eV. O this basis isomer C would be favoured, but other factors must be considered.

**Table 3:** Computational estimates of the HOMO (E_H_) and LUMO (E_L_) potentials of (EDOT-Ber^+^-EDOT)_n_ isomers in acetonitrile using the B3LYP/6-31G(d) functional.

n	A			B			C		
	E_H_	E_L_	Gap	E_H_	E_L_	Gap	E_H_	E_L_	Gap
1	− 7.845	− 5.047	2.798	− 5.774	− 2.596	3.178	− 5.808	− 2.699	3.109
2	− 5.534	− 2.932	2.602	− 5.259	− 2.654	2.605	− 5.314	− 2.772	2.542
3	− 5.516	− 2.953	2.563	− 5.218	− 2.655	2.563	− 5.319	− 2.800	2.519
4	− 5.512	− 2.991	2.521	− 5.194	2.665	2.529	− 5.323	− 2.818	2.505

Values are presented in eV and number of EDOT units either side of a berberine moiety is denoted by n.

**Table 4:** Computational estimates of the total energy and dipole moments of EDOT-Ber^+^-EDOT) isomers A, B and C in acetonitrile using the B3LYP/6-31G(d) functional.

Isomer	E_total_/Hartree	E_total_/eV	Dipole moment/D
A	0.0256	0.70	2.55
B	0.0071	0.19	9.15
C	0	0	3.57

Isomer C is taken as the energy zero.

The simulated optical spectra of isomers A, B, and C are compared in Fig. 5B. When the acetonitrile solvent model is considered, the S_1_ absorption bands are observed centred at 513 nm (A), 458 nm (B), and 466 nm (C) in acetonitrile. This suggests isomer A may be most easily oxidised assuming a correlation between electrochemical and optical gap holds.

**Figure 6:**
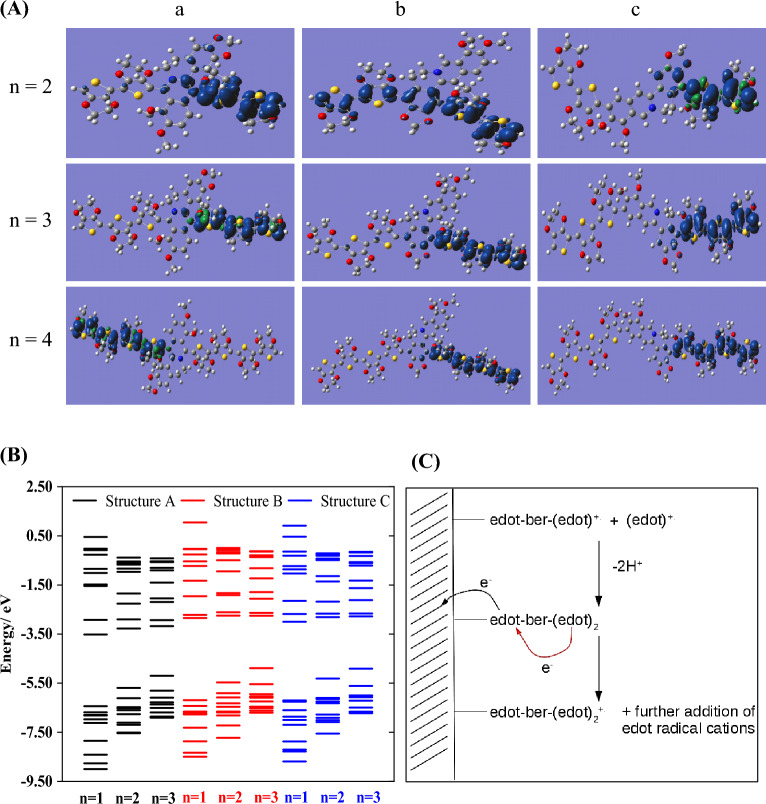
(**A**) The spin densities (blue/green) of structure (a), (b) and (c) for (EDOT)_n_(Ber^+^)(EDOT)_n_ for n = 2; n = 3 and n = 4. All calculations were performed at the geometry optimized structure using B3LYP/6-31G(d) with implicit solvation by acetonitrile. (**B**) The orbital energies of the 10 highest occupied and 10 lowest unoccupied orbitals of (EDOT)_n_(Ber^+^)(EDOT)_n_ for n = 1, 2 and 3. (**C**) Proposed mechanism of electro-copolymerization. Top. The reaction is shown as a coupling of EDOT cation radicals, one already part of a growing chain anchored to the electrode surface on the left of the diagram. Middle. Reoxidation of the newly added EDOT. Bottom. Further addition of radical cations is possible if spin density is significant on the end of the growing chain.

Figure 6B shows changes in the orbital energies of the 10 highest occupied and 10 lowest unoccupied orbitals of (EDOT)_n_(Ber^+^)(EDOT)_n_ for n = 1, 2 and 3. As expected, it highlights that the difference between the HOMO and LUMO energies decreases with increasing monomer count, resulting in a reduction of the energy difference between the HOMO and LUMO levels. This data does not however clearly favour any regioisomer and the details of the mechanism require consideration.

The HOMO, LUMO energies and the gaps for (EDOT)_n_(Ber^+^)(EDOT)_n_ species for the three structures A, B and C, for n = 1, 2, 3 and 4 are given in Table 5. The maps of spin density in these structures are given in Fig. 6A. These simulations can be interpreted in the context of Fig. 6C to provide evidence that the most promising mole ratio for copolymerization is 2:1, EDOT:Ber^+^ in isomer B.

**Table 5:** The energies (referenced to vacuum) of the HOMO and the LUMO of (EDOT)_n_-Ber^+^-(EDOT)_n_ isomers where EDOT is ethylenedioxythiophene and Ber^+^ is berberine cation, with the respective energy gaps (E_g_).

	E_HOMO_/eV	E_LUMO_/eV	E_g_/eV
Structure A			
(EDOT)-(Ber^+^)-(EDOT)	− 6.4374	− 3.5179	2.9195
(EDOT)_2_-(Ber^+^)-(EDOT)_2_	− 5.6926	− 3.2727	2.4199
(EDOT)_3_-(Ber^+^)-(EDOT)_3_	− 5.2001	− 3.1777	2.0224
Structure B			
(EDOT)-(Ber^+^)-(EDOT)	− 6.1914	− 2.8457	3.3457
(EDOT)_2_-(Ber^+^)-(EDOT)_2_	− 5.4700	− 2.7467	2.7233
(EDOT)_3_-(Ber^+^)-(EDOT)_3_	− 4.8907	− 2.7532	2.1375
Structure C			
(EDOT)-(Ber^+^)-(EDOT)	− 6.2036	− 3.0003	3.2033
(EDOT)_2_-(Ber^+^)-(EDOT)_2_	− 5.3113	− 2.8074	2.5039
(EDOT)_3_-(Ber^+^)-(EDOT)_3_	− 4.9089	− 2.7780	2.1309

Figure 6C shows a schematic illustration of the copolymerization of Ber^+^ and EDOT at the end of a growing polymer chain that is anchored (on the left of the diagram) to the electrode surface. Coupling of new radicals to the growing chain is feasible if there is significant spin density at the end of the chain far from the electrode. Figure 6A shows the spin densities for the one-electron oxidation products of species (EDOT)_n_(Ber^+^)(EDOT)_n_ for n = 2, 3 and 4 and isomers A, B and C of Fig. 5A In all cases, except one, the spin density is localized on one side of the Ber^+^ unit. This suggests that these systems will not readily polymerise because the growing ends of the polymer chain remote from the electrode will not acquire spin density to couple to further radical cations. However, isomer B with n = 2 does show the spin density on both sides of the Ber^+^ unit. We interpret this effect in terms of a mismatch between the energies of the EDOT and Ber^+^ orbitals – as the EDOT chain lengthens, the orbital energies change. This series of calculations suggests that the ratio EDOT:Ber^+^ should not be too large for successful copolymerisation and that isomer B is the most likely product.

### Experimental copolymerization of berberine and EDOT

**Figure 7:**
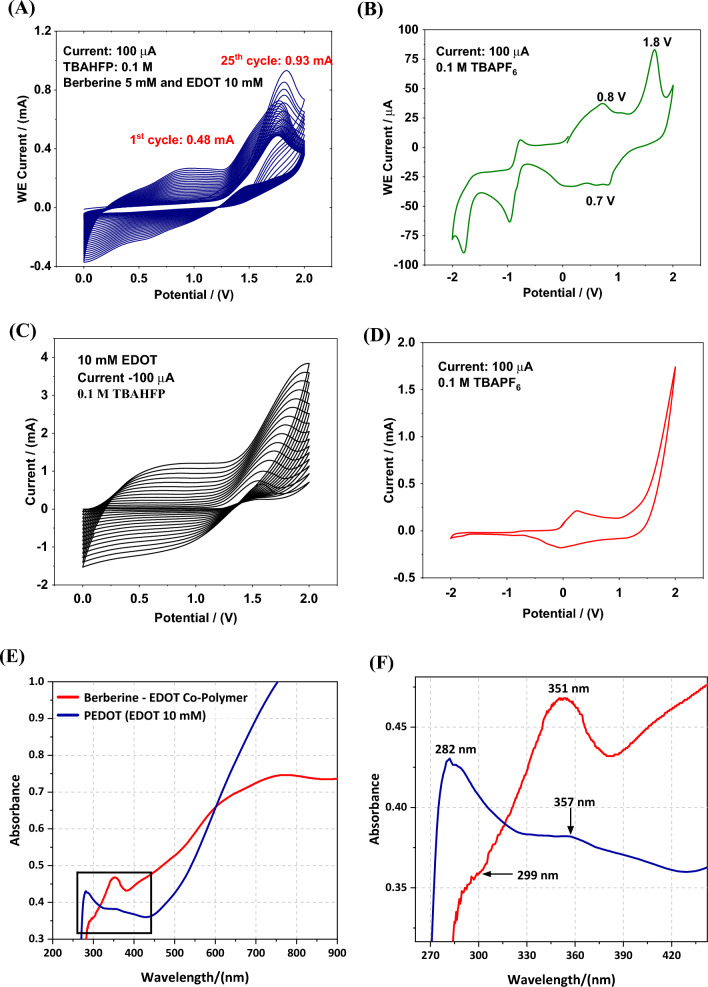
(**A**) Cyclic voltammograms (CVs) of 5.0 mM berberine cation (Ber^+^) and 10.0 mM EDOT in 0.10 M TBAPF_6_ solution between 0 and 2.0 V potential range, and (**B**) CV of the polymer in 0.10 TBAPF_6_ solution (neat background electrolyte without the monomers) between − 2.0 V–2.0 V potential range. (**C**) CVs of 10.0 mM PEDOT in 0.10 M TBAPF_6_ in acetonitrile solution between 0 V–2.0 V potential range. (**D**) CV of the polymer in 0.10 TBAPF_6_ acetonitrile solution between − 2.0 V–2.0 V potential range. (**E**) UV–Visible absorption spectra of Ber^+^-EDOT co-polymer and PEDOT. (**F**) Spectra in (**E**) in the range from 270 to 450 nm showing π $\to$ π* and n $\to$ π* transitions.

Figure 7 illustrates the CVs acquired during the attempted electro-polymerization process of Ber^+^ with EDOT at 1:2 molar ratio. Figure 7A exhibits the repetitive CVs that depict the successive electro-polymerization leading to the formation of a polymer film on the surfaces of GC electrodes. The increasing current observed in consecutive CVs in Fig. 7A indicates the deposition of conducting polymer films onto the GC working electrode surface. In Fig. 7B, the CV obtained for the suspected polymer in the BGE without monomers is displayed. The CVs of the polymers shown in Fig. 7B provide clear evidence of the incorporation of the EDOT and Ber^+^ in the copolymer where the presence of EDOT is indicated by an oxidation peak at approximately + 0.80 V during the forward scan, followed by a corresponding reduction peak at around + 0.70 V during the reverse scan. Additionally, an oxidation peak at approximately + 1.8 V is observed for the first oxidation step of Ber^+^.

The CVs of the PEDOT in Fig. 7C and D show their typical capacitive behaviour if recorded within 0 V to + 1.0 V with typical oxidation peak centred at + 0.25 V in the forward scan with its reduction peak at − 0.41 V in the reverse scan. There are hardly any currents in the negative potential range from – 1.0 V to – 2.0 V wrt Ag/AgCl/KCl(sat.) electrode. PEDOT gets over-oxidized above + 1.50 V as we see from the sharp rise in currents above + 1.53 V. This CV is typical of PEDOT as it is conducting at positive potentials due to the formation of polarons and biplarons. When the potential is reversed, it takes time to neutralise the charge carriers formed and currents are observed down to – 0.7 V beyond which it is not conducting as there are no appreciable currents.

The absorption spectra, shown in Fig. 7E, have distinct differences. Both show absorption in the entire region 270 nm to 900 nm range that we could measure indicating that the materials are not simple molecules or oligomers. The fact that both materials show high absorptions in the entire range indicates that they are electronically conducting polymers. PEDOT shows π $\to$ π* transition at 282 nm while the same of the copolymer is centred at 299 nm with a 17 nm bathochromic shift. The lowering of band gap due to the localisation of HOMO and LUMO levels at D and A units, respectively, gives this bathochromic shift of the π $\to$ π* transition as shown in Fig. 7F. Interestingly, the n $\to$ π* transition of the copolymer appearing at 351 nm shows a small hypsochromic shift of 6 nm from that of PEDOT (357 nm) though the maximum of the absorption band is difficult to locate. If there is such a small hypsochrmic shift it may be due to the decrease of n-electron concentration in the conjugated chain due to the presence of some Ber^+^ units instead of EDOT units in the copolymer. However, note that EDOT has 8 non-bonded electrons in its ethylenedioxy moiety while Ber^+^ has 16 of them in methylenedioxy and methoxy groups. However, the conjugated chain has electron deficient N^+^ making the n-electron density calculation difficult. The overall effect could be the reason for small difference observed in $n \to$ π* transitions of the two materials. Additionally, the low energy polaron and bipolaron bands are clearly observed in both materials.

**Figure 8:**
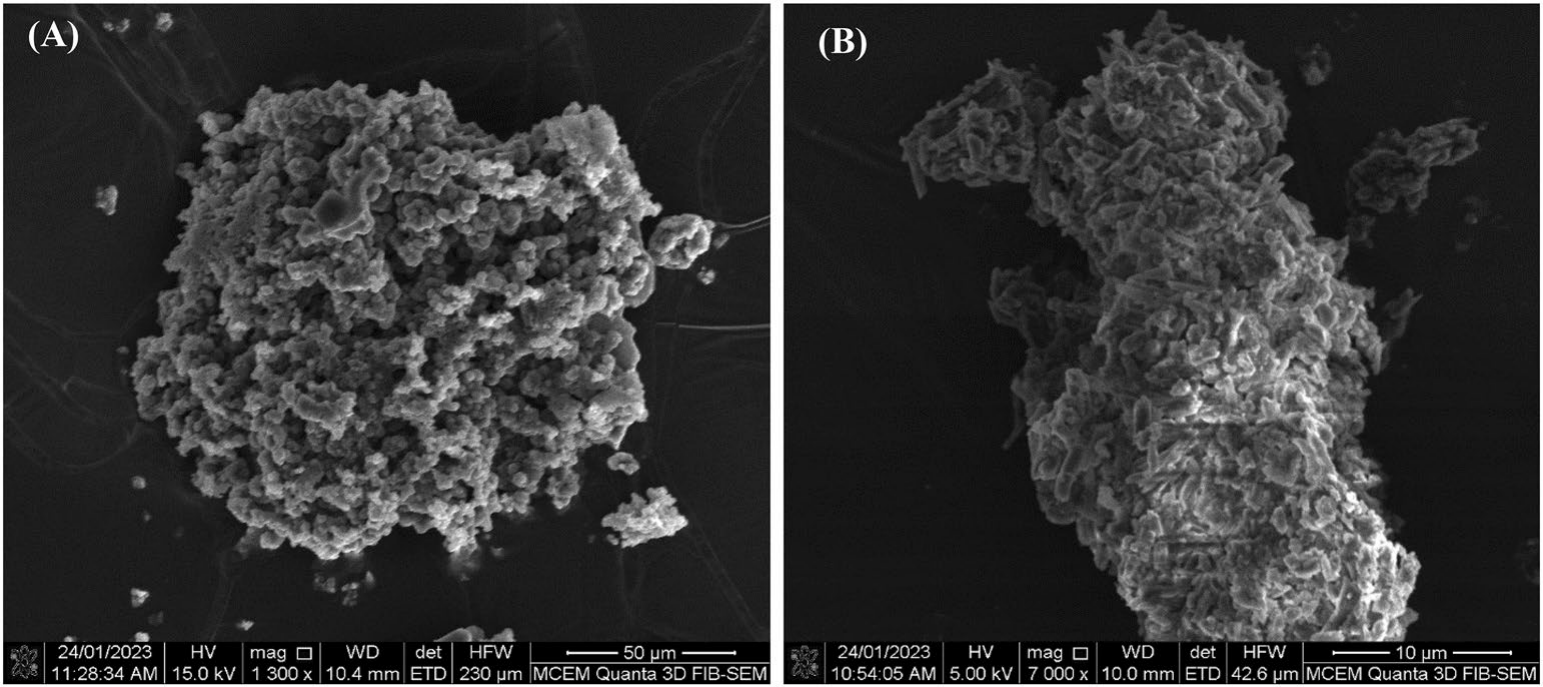
Scanning electron microscopy images (SEM) of (**A**) Berberine with magnification 1300x (**B**) the suspected polymer with magnification 7000x.

Electrochemical analysis provided initial insights into the polymer composition, which were further supported by SEM–EDX analysis. SEM images (Fig. 8) reveal the morphology of the polymer films on FTO glass. In comparison, SEM images of bare FTO showed grain structures of FTO, which facilitated epitaxial polymer growth and resulted in a thermodynamically favoured cloud-like structure. The atomic ratios obtained from SEM–EDX analysis were complex; however, elemental mapping at the macroscopic scale exhibited acceptable levels of carbon, nitrogen, oxygen, and sulphur, providing evidence in support of copolymerization.

Tables 6 and 7 present the theoretically calculated and experimentally obtained atomic percentages of berberine cation and the (EDOT-Ber^+^-EDOT)_n_ copolymer. These tables provide detailed information regarding the atomic composition of the copolymer, further supporting the successful formation of the desired polymer structure.

**Table 6:** Theoretically calculated and experimental average atomic percentages of berberine chloride where the experimental atomic averages were obtained by energy dispersive X-ray (EDX) analysis of the scanning electron microscopic (SEM) images taken from different sites of the images.

Berberine chloride	C%	H%	N%	O%	Cl%
Theoretical atomic%	45.45	40.90	2.27	9.09	2.27
Theoretical atomic% excluding H	76.92		3.85	15.39	3.85
Experimental	83.60		2.45	11.33	2.05

**Table 7:** Theoretically calculated and experimental average atomic percentages of the suspected polymer obtained by EDX analysis of the SEM images taken from different sites of the images.

Polymer atomic percentages	C%	H%	N%	O%	S%	P%	F%
Theoretical (EDOT-Ber^+^ PF_6_^–^)_n_	35.29	42.65	1.47	8.82	1.47	1.47	8.82
Theoretical (EDOT-Ber^+^ PF_6_^–^)_n_ excluding H	61.54		2.56	17.94	2.56	2.56	15.38
Theoretical (EDOT-Ber^+^PF_6_^–^EDOT)_n_	32.94	47.05	1.17	9.41	2.35	1.17	7.05
Theoretical (EDOT-Ber^+^PF_6_^–^EDOT)_n_ excluding H	60.87		2.17	17.39	4.34	2.17	13.04
Theoretical (EDOT-Ber + PF_6_^–^EDOT^+^ PF_6_^–^)_n_	30.43	43.47	1.09	8.70	2.17	2.17	13.04
Theoretical (EDOT-Ber + PF_6_^–^EDOT^+^ PF_6_^–^)_n_ excluding H	52.83		1.88	15.09	3.77	3.77	22.64
Experimental	63.45		1.58	16.43	4.47	2.47	12.26

Data in Table 6 show some differences between the theoretical and empirical atomic percentages of Ber^+^Cl^-^. This is because the SEM-EDAX is incapable of measuring element H and hence calculating atomic percentages with respect to the measured elements. When the atomic percentages were calculated excluding H atoms a somewhat closer agreement can be observed. However, empirical results show good agreement for N and Cl atomic percentages when H is included.

As can be seen from the data, the presence of C, N, O, S, P and F suggests that both EDOT and Ber^+^PF_6_^–^ are incorporated in the polymer. Since the polymer was grown from a solution containing large excess of TBAPF_6_, it is possible to incorporate PF_6_^–^ ion as the counter ion and the chloride ion remaining in the solution. As explained, SEM-EDAX is incapable of measuring element H and hence the atomic percentages determined are based on those measured. When we consider the possibility for oxidation of Ber^+^ forming structures given in Fig. 3, at positive potentials, the polymer may contain these species also thus increasing atomic percentage of O. Since the polymer is electronically conducting there may be more than one counter ions ([PF_6_]^–^) in the repeat unit as EDOT may exist as EDOT^.+^ in it. Considering these possibilities theoretical atomic percentages were calculated for all three cases with and without including H atoms. The best correlation is obtained for Theoretical (EDOT-Ber^+^PF_6_^–^EDOT)_n_ excluding H. It is, therefore, possible that the 1:2 stoichiometry is favoured in the polymer because EDOT is the one that is easiest to oxidize forming cation radical EDOT^.+^ (at around + 0.8 V), and it can then combine with a radical on the growing polymer chain. However, Ber^+^ is difficult to oxidize to form Ber^2+.^radical. The possible way forward for propagation of the reaction is, therefore, to react EDOT^.+^-Ber^+^ with another EDOT^.+^ forming EDOT^.+^- -Ber^+^- EDOT^.+^ as the repeat unit giving a copolymer with EDOT:Ber^+^ stoichiometry of 1:2 as suggested in Fig. 6.

**Figure 9:**
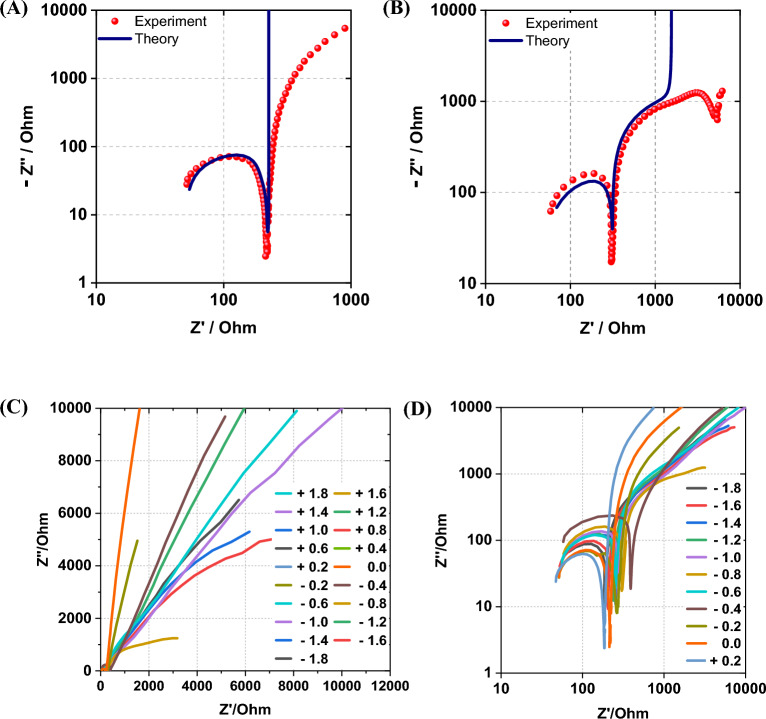
Impedance spectra for poly(Ber^+^-EDOT) films over the frequency range 1 MHz–1 Hz. The electrolyte was 0.1 mol dm^–3^ TBAPF_6_. (**A**) dc potential of 0.0 V; (**B**) dc potential of − 0.8 V. The symbols are experimental data, and the solid line is the least squares fit of the impedance of the equivalent circuit of Fig. 10A. (**C**) Nyquist plots of range − 1.8 V–1.8 V. (**D**) Experimental dc potentials of range − 1.8 V–0.2 V.

Impedance spectra were recorded at different applied potentials to evaluate the electronic and ionic conductivity of poly(Ber^+^-EDOT). Nyquist [–Im(Z) vs Re(Z)] plots obtained at selected potentials of − 0.8 V and 0.0 V for the 2:1 co-polymer is given in Fig. 9. The full dataset is shown in the SI. The impedance spectra are presented with logarithmic axes scales in order to display clearly their main features; a high frequency semi-circle and a low frequency region in which the impedance is either capacitive (Fig. 9A) or has the form of a flattened semi-circle (Fig. 9B). We analysed these spectra using the dual-rail transmission line model^28^ shunted by a capacitor (*C*_*2*_) to describe the low frequency region and a parallel *R*_*ct*_*C*_*1*_ circuit to describe the high frequency region (Fig. 9A).

**Figure 10:**
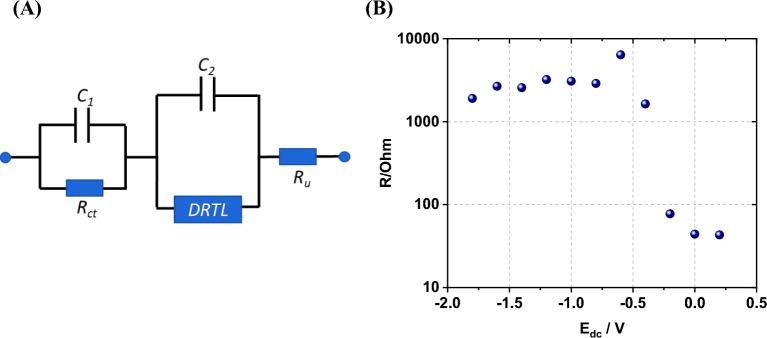
(**A**) Equivalent circuit used to fit the impedance spectra of poly(Ber+-EDOT). DRTL indicates a dual-rail transmission line with resistive rails (common resistance, R) representing the electronic and ionic conduction pathways and distributed capacitance (value not shown). (**B**) lnR against dc potential. Ru is the uncompensated solution resistance and was found to be of the order of 50 Ohm.

The impedance spectrum defined by the equivalent circuit of Fig. 10A was fitted to the experimental data by the complex least squares method. Three main regions are observed in these spectra and denoted (i), (ii) and (iii) in the discussion. (i) a semicircle at large frequency (R about 200 Ω); (ii) an incomplete semicircle at lower frequency and (iii) in the equivalent circuit (blue) the impedance eventually becomes capacitive at the very lowest frequency, but the data does not.

We observed that at potentials above 0.2 V, the quality of the fit is poor; this is the region of the cyclic voltammogram (Fig. 7B) in which the polymer is p-doped, and its impedance is small. At positive potentials, there is also the possibility of oxidation of berberine^8^ and the simple equivalent circuit of Fig. 10A cannot model such a faradaic process. The fit of this model to the data for E < 0.2 V is satisfactory except at very low frequencies (iii) where the impedance spectrum is strongly influenced by background faradaic processes and deviates from the predicted pseudo-capacitive behaviour of any model based on a finite thickness polymer film and no background faradaic process. Nevertheless, we can fit the two main features of interest (i), (ii) and extract an effective charge transfer resistance (*R*_*ct*_) for the high-frequency semicircle and the resistance of the rails of the transmission line (*R*) for the low-frequency data.

The resistance *R*_*ct*_ extracted from the high frequency semi-circle is relatively insensitive to the applied potential, with no clear trend and a value of 210 ± 63 Ohm almost independent of potential. *R*_*ct*_ is sensitive to the background electrolyte concentration and at -0.6 V it has a value of 207 Ohm, 327 Ohm and 416 Ohm in concentrations of 0.1, 0.05 and 0.03 mol dm^-3^ TBAPF_6_. On this basis, we do not assign *R*_*ct*_ as a true charge transfer resistance but suggest that it is an effective resistance associated with the high-frequency response of the cell and electronics – the associated *R*_*ct*_*C*_*1*_ time constant is of the order of 0.5 μs.

The second, lower frequency, incomplete semi-circle (region (ii)) contains information on charge transport in the polymer, which we represent by a transmission line element (Fig. 10A). The dual-rail transmission line is characterized by the resistances of the two rails, representing the electronic and ionic conduction and the distributed capacitance^29^. We found that a common value of resistance (*R*) for both rails was sufficient to fit the experimental data. The apparent equality of the resistance of electronic and ionic resistances has also been observed by previous workers^30^. Figure 10B shows the variation of *R* with applied potential over the range 0.2 V to − 1.8 V. There is a clear increase in R from a value of about 40 Ohm to one of about 3 kOhm as the potential becomes more negative than − 0.2 V. This is the region of the cyclic voltammogram (Fig. 7B) at which the polymer is nominally undoped. We estimate the transition potential to be about − 0.3 ± 0.05 V. In the case of pure poly(EDOT)^31^, the impedance spectrum shows a large semicircle in the undoped region whose diameter increases by several orders of magnitude as the potential becomes more negative. In contrast, although *R* increases below − 0.2 V in poly(Ber^+^-EDOT) it reaches an approximately constant value of the order of 3 kOhm from − 0.4 V down to the most negative potentials studied. We interpret this non-zero conductivity in the absence of oxidative doping as evidence of some self-doping of poly(Ber^+^-EDOT) because of the presence of its electron-accepting isoquinolinium moiety.

## Conclusion

We find that direct polymerization of berberine does not occur, in agreement with calculations and previous works on the oxidation chemistry of berberine cation^12^. However, copolymerization of berberine from solutions containing mixtures of EDOT and berberine is possible. The cyclic voltammetry of the polymerization media shows the expected growth in current as the polymer film forms on the electrode surface. Voltammetry of the film in the absence of monomer shows both the broad waves expected of EDOT doping/undoping and additional peaks at positive and negative potentials which are characteristic of berberine^8^. DFT calculations suggest that the most likely structure of the copolymer is that formed by attaching EDOT units via their 2 or 5 positions to C atoms 15 and 18 of the berberine cation for (EDOT)_n_-Ber^+^-(EDOT)_n_ type polymers and C atoms 1 and 15 for (EDOT-Ber^+^-EDOT)_n_ type polymers. Impedance spectroscopy provides evidence of some residual conductivity (“self-doping”) in the potential region in which the polymer is nominally undoped according to the cyclic voltammetry.

### Supplementary Information

Supplementary Information.

## Data Availability

The datasets used and/or analyzed during the current study are available from BRH, HMNPG upon reasonable request.
