# Supplementary material for: Computational analysis and experimental verification of donor–acceptor behaviour of berberine, and its co-oligomers and co-polymers with ethylenedıoxythıophene
Source: Sci Rep. 2023 Nov 18;13:20186. doi: 10.1038/s41598-023-47541-7 (PMC10657409; doi:10.1038/s41598-023-47541-7)
Supplement: Supplementary file 1 — Supplementary Information. [file 41598_2023_47541_MOESM1_ESM.pdf]

Ber$^+$ Frequency Analysis.

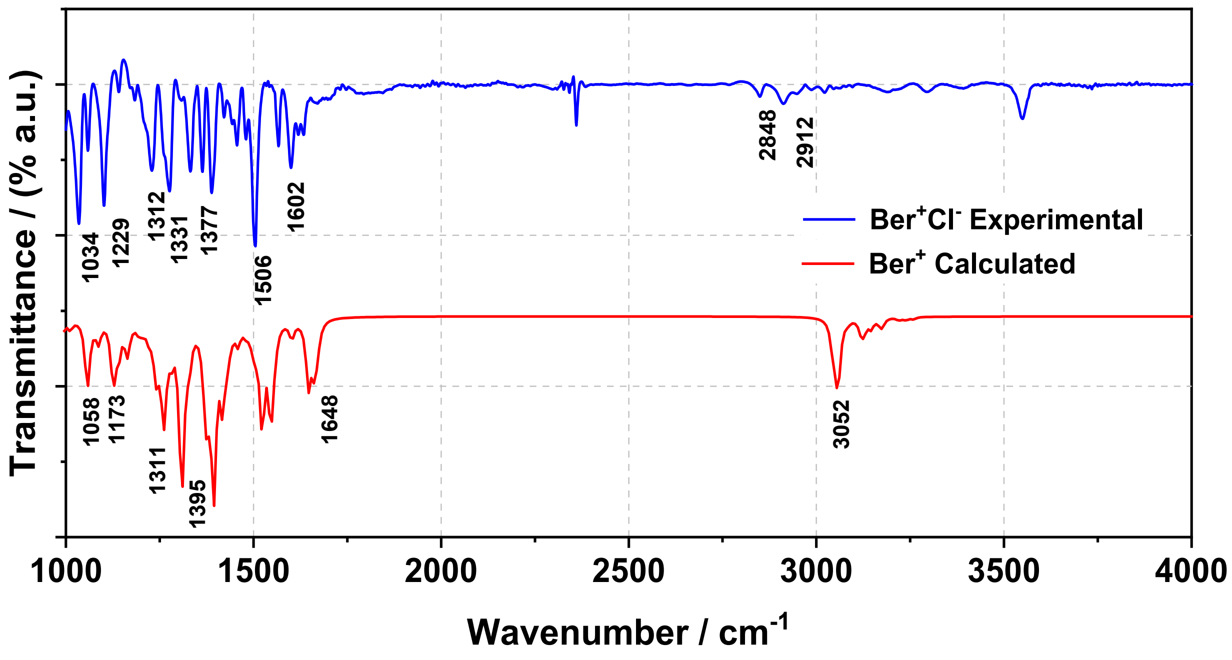

SI Figure 01: FT-IR spectra of Ber$^+$ calculated (red), and experimental (blue).

SI Table 01: FT-IR spectra analysis of Figure 01.

Functional group/bond	Bonding nature	Wavenumber / cm^{-1}	
		Calculated	Experimental
O-C-O	C-O Stretching	1058	1034
C-O-CH_3	C-O Stretching	1173	1229
-C-N-	Stretching	1311	1312
Aromatic -C-H	Bending	1305	1331
Aromatic -C-C-	Stretching	1395	1377
Aromatic =C-H	C-H Bending	1329 - 1418	1390 -1453
C-O-CH_3	C-H Bending	1500 - 1514	1506
Aromatic C=C	Stretching	1602-1673	1600-1634
C=N	Stretching	1648	1602
O-C-H_3	Stretching	3045<br>3052	2848<br>2912

FT-IR analysis of resultant solution from potential range (1.4 – 2.0) V after the oxidation of $Ber^{+}Cl^{-}$.

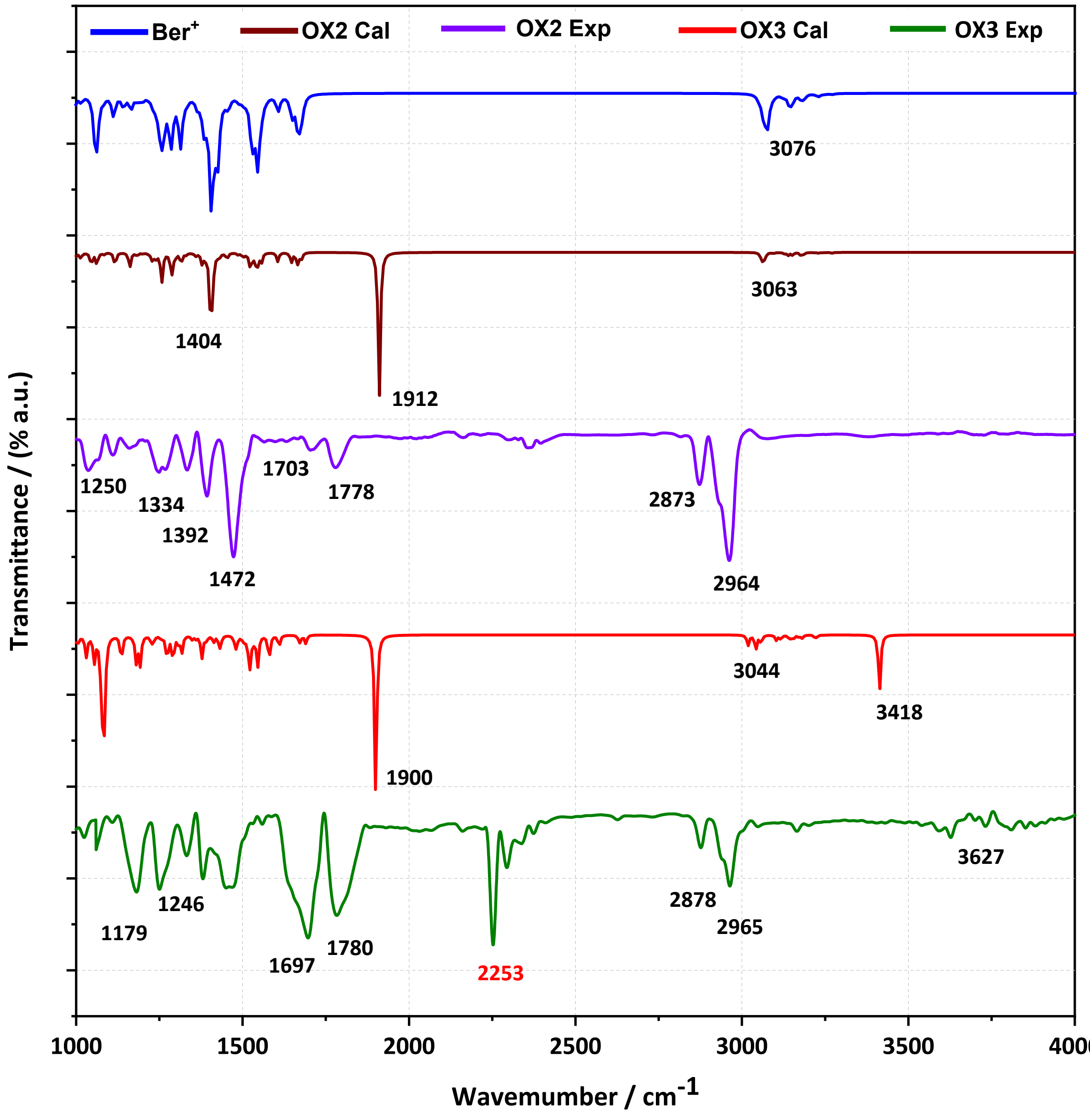

SI Figure 02: FT-IR spectra of oxidized product isolated at 1.65 V potential. OP9: Calculated IR spectrum of possible oxidation product 9 (blue), and OX3: Experimental oxidation product (green).

C≡N stretching vibration of the acetontrile shows the IR band at 2253 cm^{-1} OX3 Exp spectrum.

SI Table 02: FT-IR spectra analysis of SI Figure 02.

Functional group/bond	Bonding nature	Wavenumber / cm^{-1}			
		Oxidation product 2 (OX2)		Oxidation product 3 (OX3)	
		Calculated	Experimental	Calculated	Experimental
-C-O-CH_3	C-O Stretching	1181<br>1183	1250	1226<br>1255	1246
-C-N-	Stretching	1359	1334	1348	1332
-C-H	Bending	1404	1392	1391-1412,<br>1496	1446-1475
=C-H	Stretching	1486	1472	1520	1556
-C=C-	Stretching	1667	1778	1666-1690	1697
C=O	Stretching	1911	1778	1900	1780
-OH	Stretching	-	-	3416	3627
-O-CH_3	C-H Stretching	3063<br>3071	3076	2878<br>2965	3044<br>3060

Theoretical IR analysis of possible oxidation products.

OX2

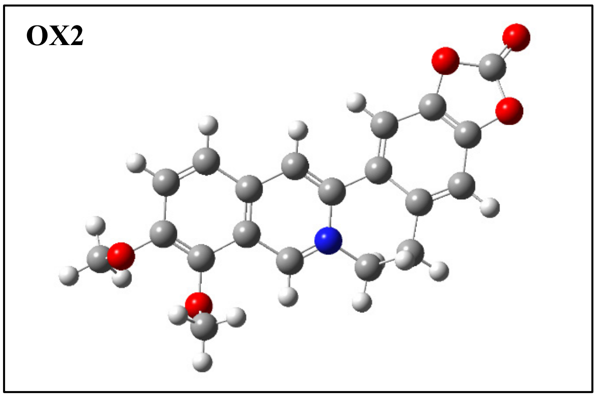

OX3

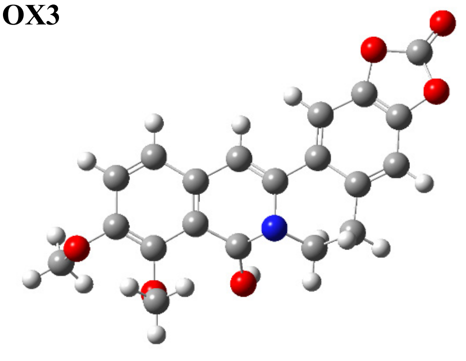

OP1

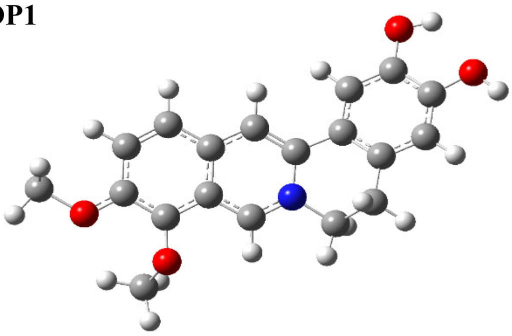

OP2

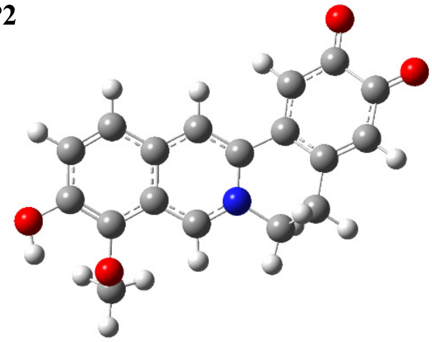

OP3

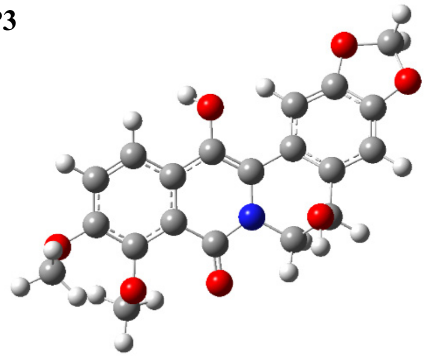

OP4

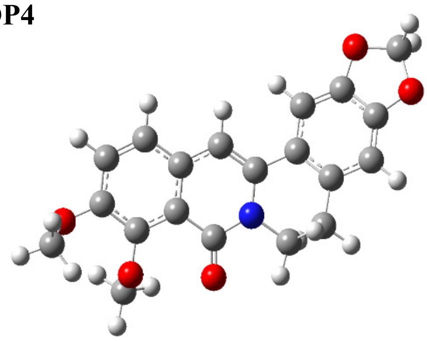

OP5

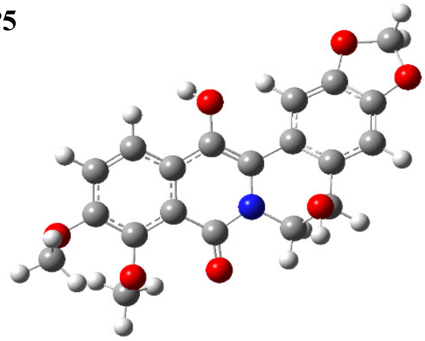

OP6

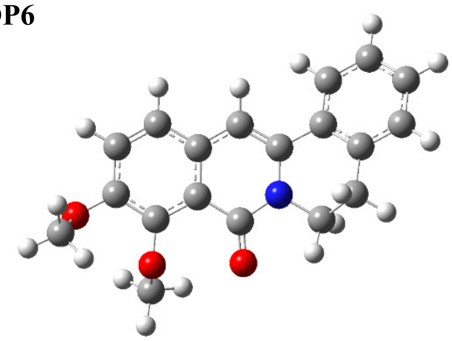

OP7

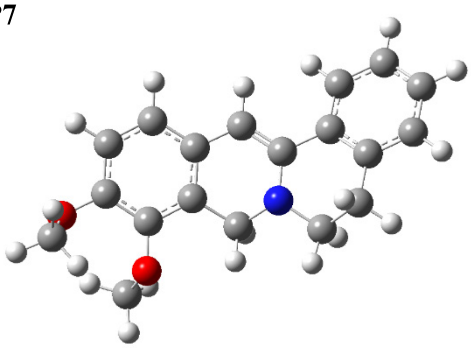

OP8

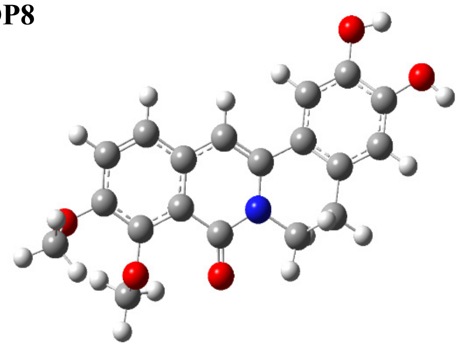

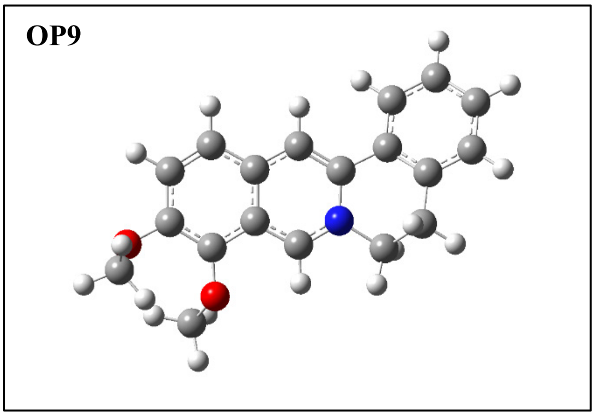

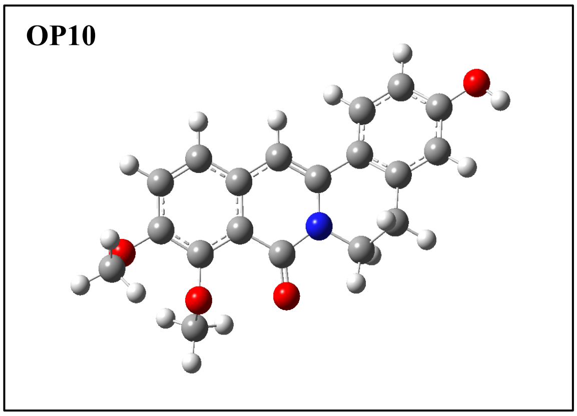

SI Figure 03: Geometry optimized possible oxidation products using B3LYP/6-31G(d) basis set and acetonitrile solvent medium.

OX2

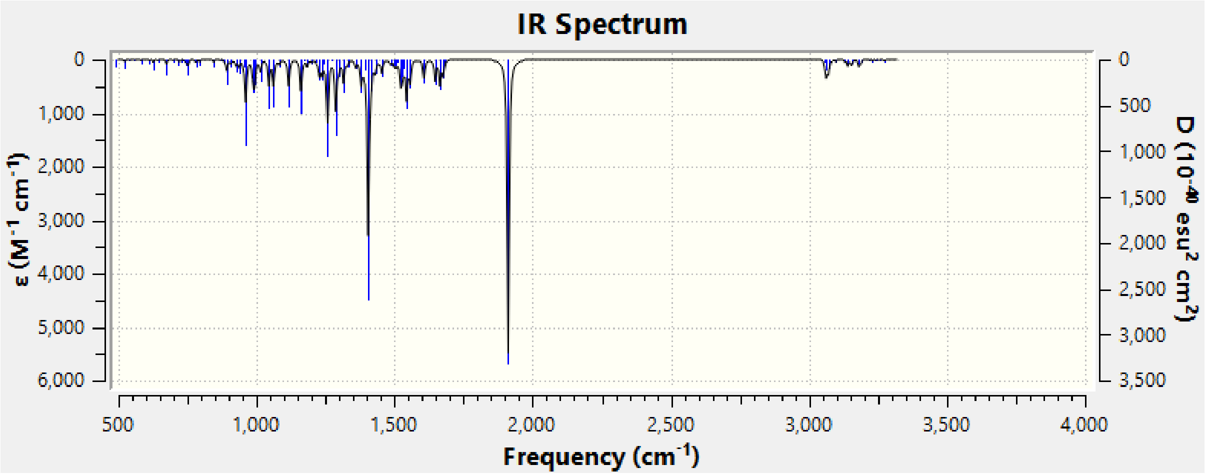

OX3

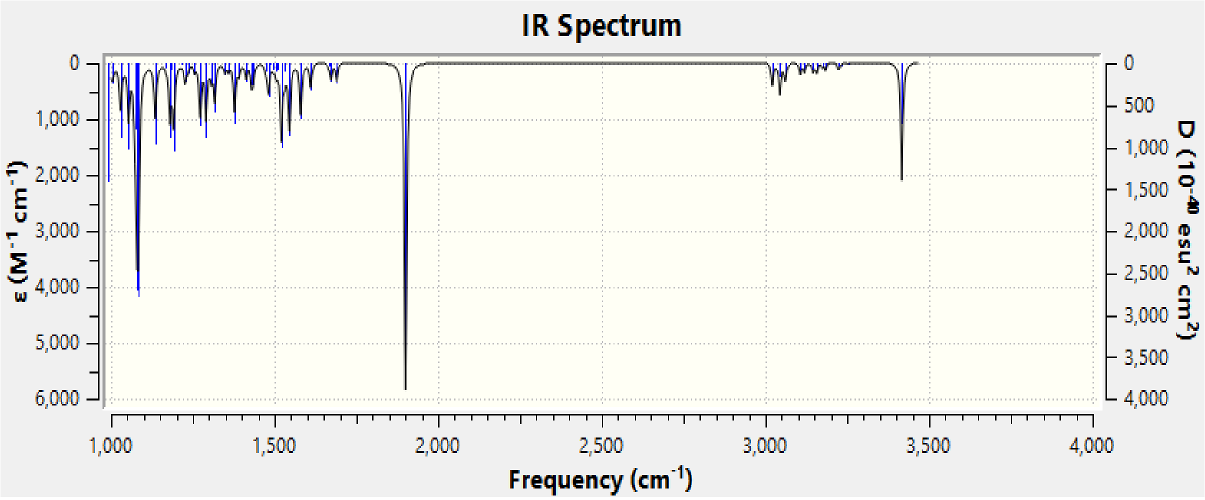

OP1

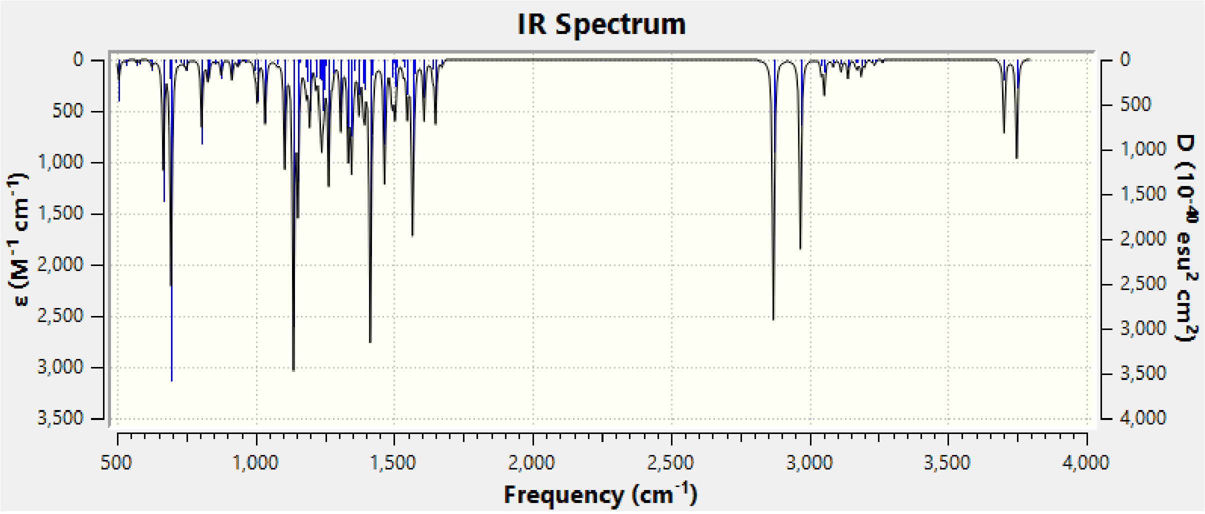
IR Spectrum
ε (M-1 cm-1)
D (10-40 esu2 cm2)
Frequency (cm-1)

OP2

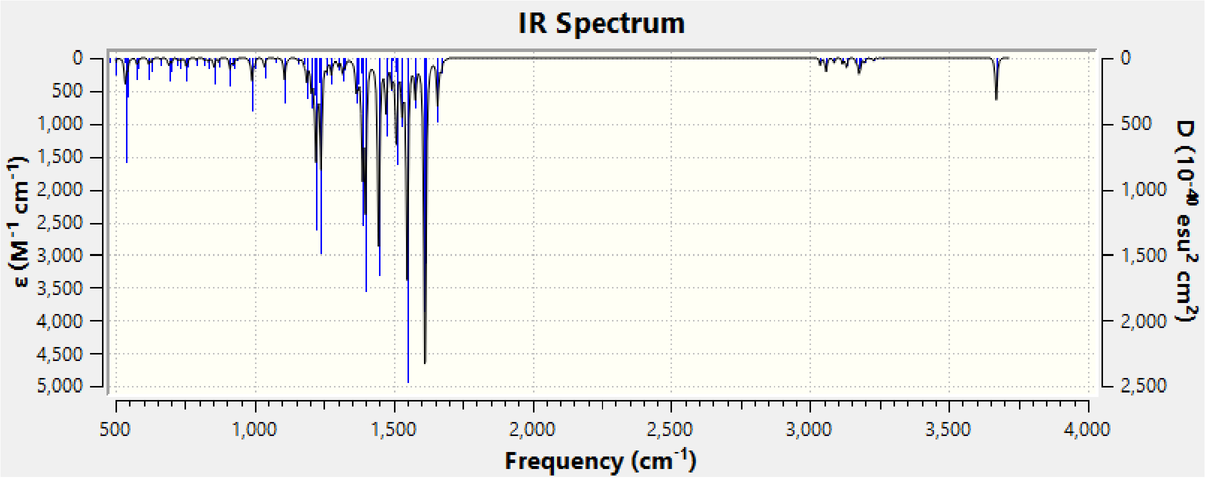
IR Spectrum
ε (M-1 cm-1)
D (10-40 esu2 cm2)
Frequency (cm-1)

OP3

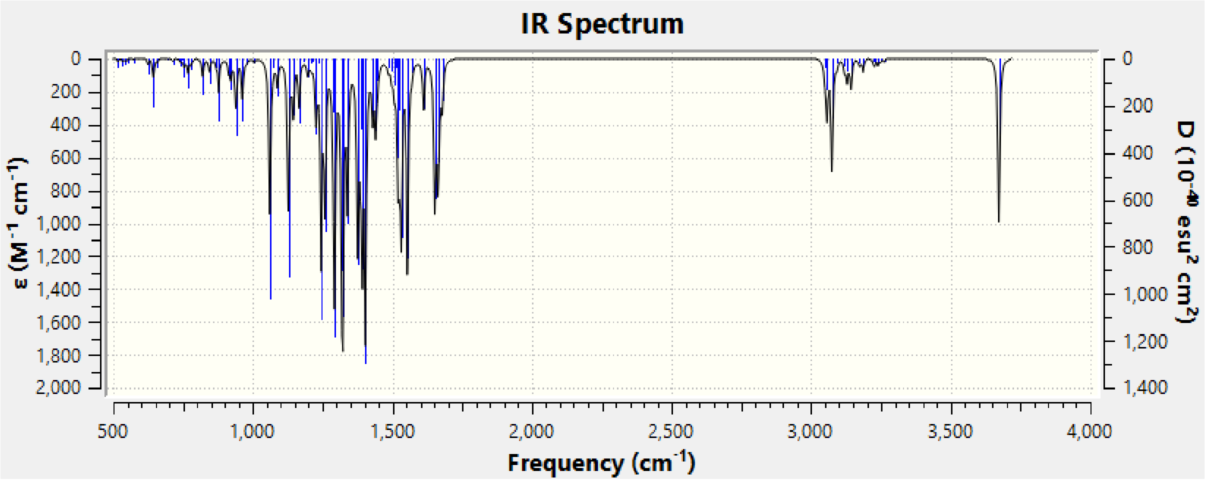
IR Spectrum
ε (M-1 cm-1)
D (10-40 esu2 cm2)
Frequency (cm-1)

OP4

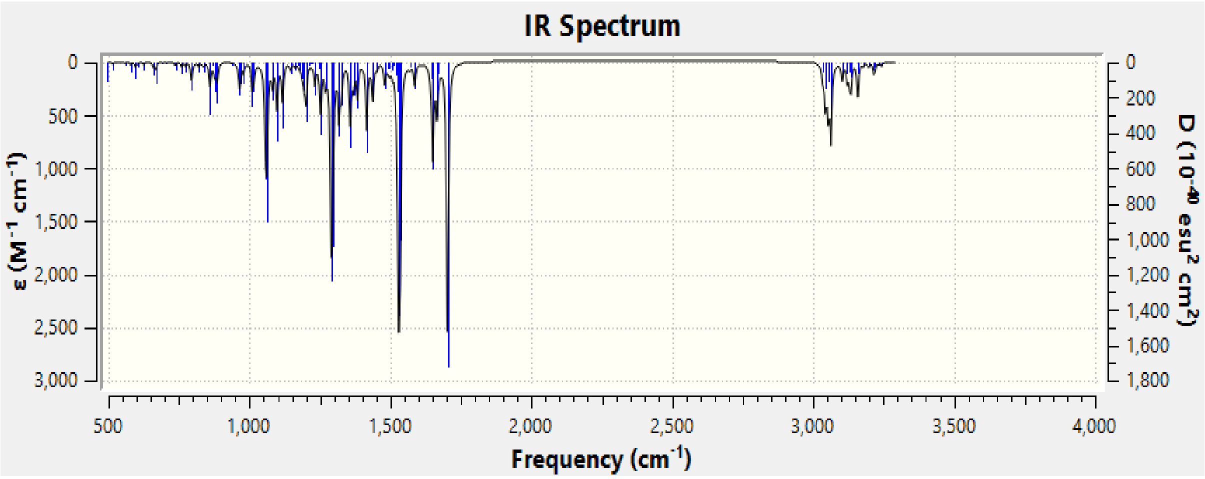
IR Spectrum
ε (M⁻¹ cm⁻¹)
0
500
1,000
1,500
2,000
2,500
3,000
D (10⁻⁴⁰ esu² cm²)
0
200
400
600
800
1,000
1,200
1,400
1,600
1,800
500
1,000
1,500
2,000
2,500
3,000
3,500
4,000
Frequency (cm⁻¹)

OP5

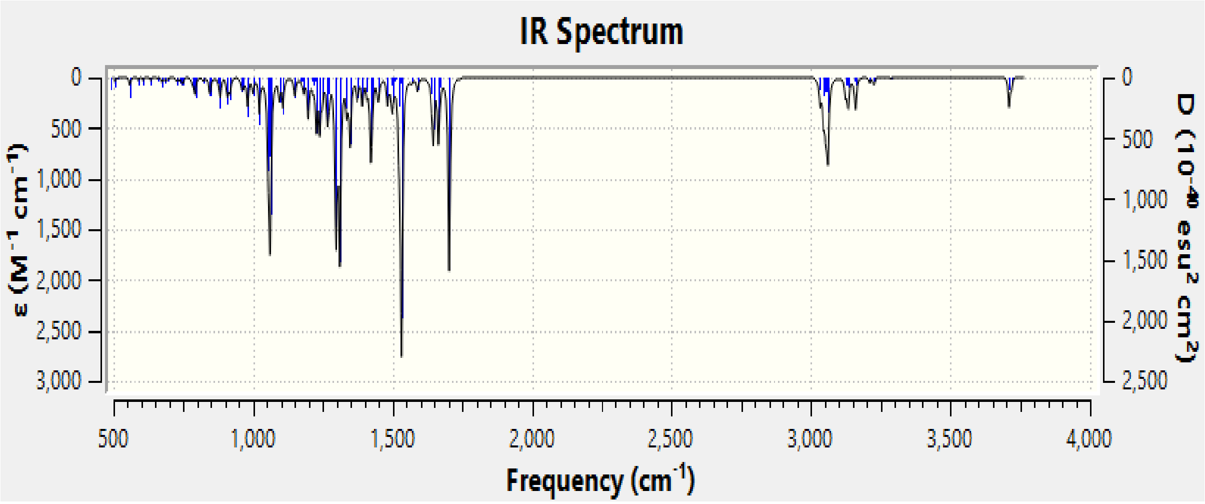
IR Spectrum
ε (M⁻¹ cm⁻¹)
0
500
1,000
1,500
2,000
2,500
3,000
D (10⁻⁴⁰ esu² cm²)
0
500
1,000
1,500
2,000
2,500
500
1,000
1,500
2,000
2,500
3,000
3,500
4,000
Frequency (cm⁻¹)

OP6

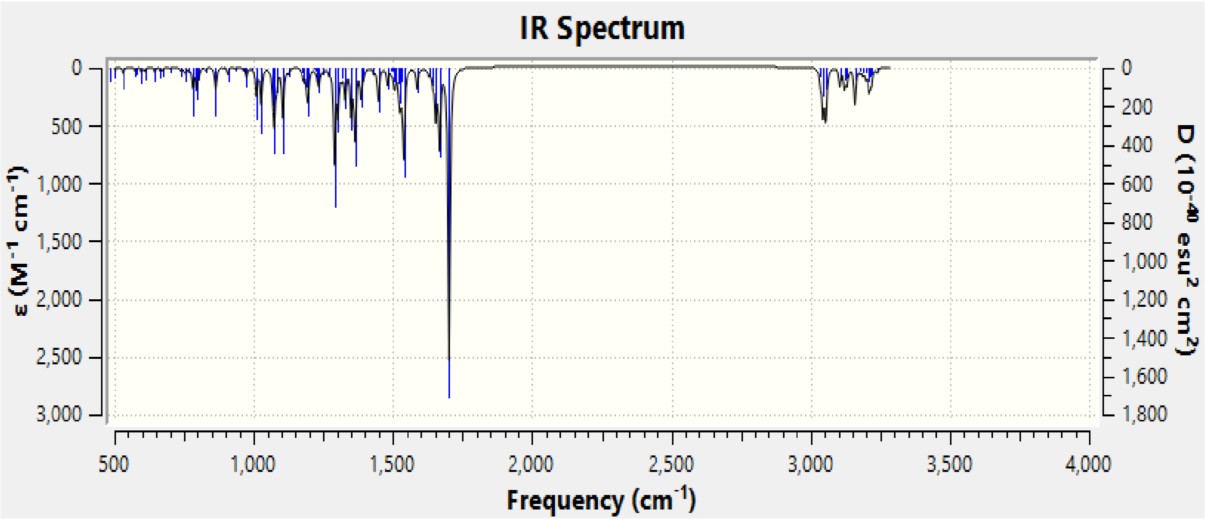
IR Spectrum
ε (M⁻¹ cm⁻¹)
0
500
1,000
1,500
2,000
2,500
3,000
D (10⁻⁴⁰ esu² cm²)
0
200
400
600
800
1,000
1,200
1,400
1,600
1,800
500
1,000
1,500
2,000
2,500
3,000
3,500
4,000
Frequency (cm⁻¹)

OP7

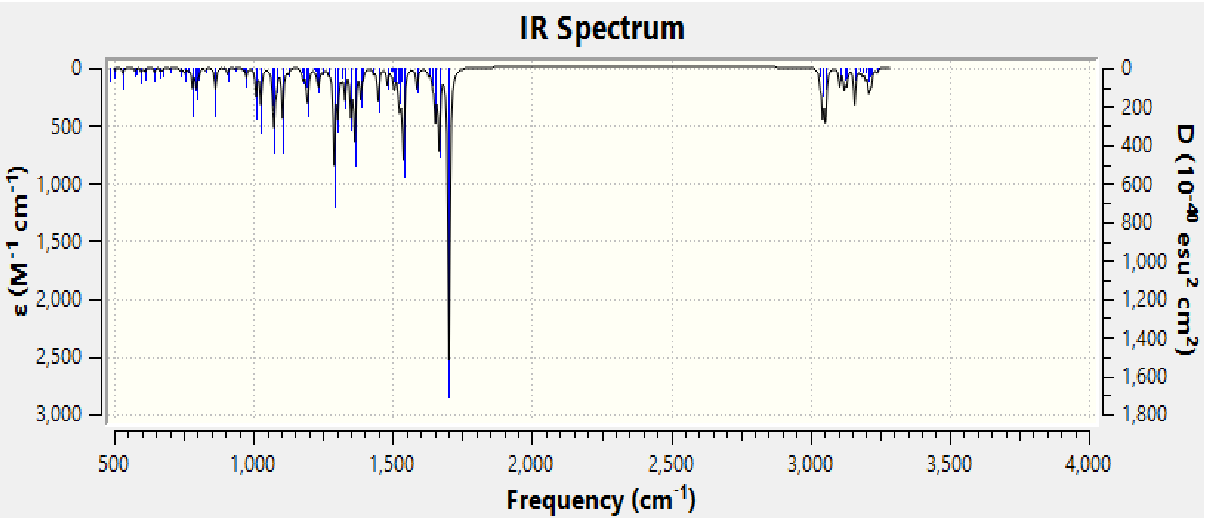
IR Spectrum
ε (M⁻¹ cm⁻¹)
0
500
1,000
1,500
2,000
2,500
3,000
D (10⁻⁴⁰ esu² cm²)
0
200
400
600
800
1,000
1,200
1,400
1,600
1,800
500
1,000
1,500
2,000
2,500
3,000
3,500
4,000
Frequency (cm⁻¹)

OP8

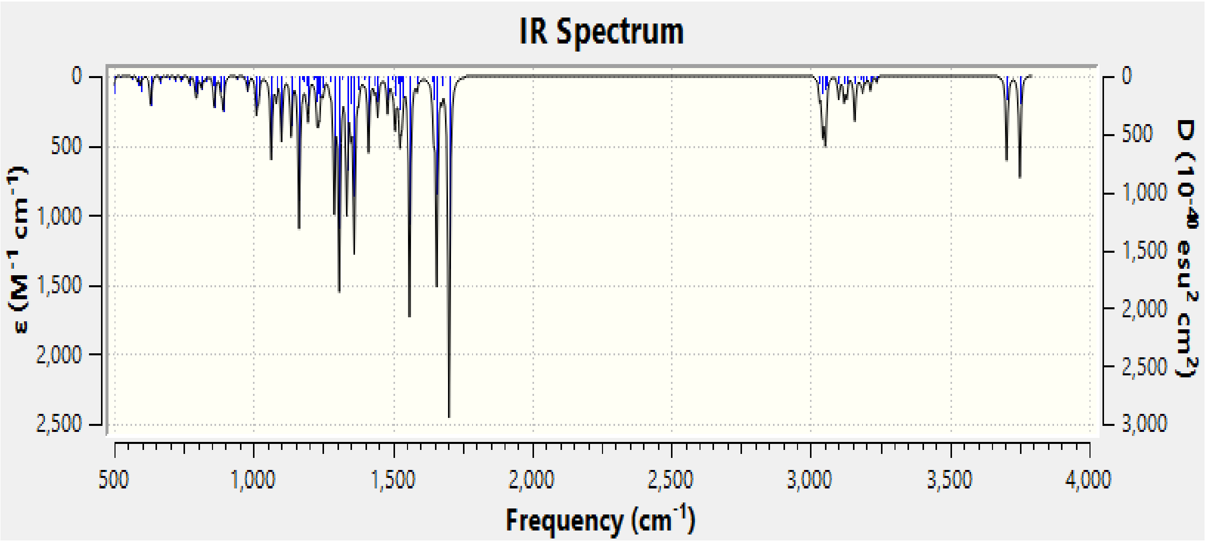
IR Spectrum
ε (M⁻¹ cm⁻¹)
0
500
1,000
1,500
2,000
2,500
D (10⁻⁴⁰ esu² cm²)
0
500
1,000
1,500
2,000
2,500
3,000
500
1,000
1,500
2,000
2,500
3,000
3,500
4,000
Frequency (cm⁻¹)

OP9

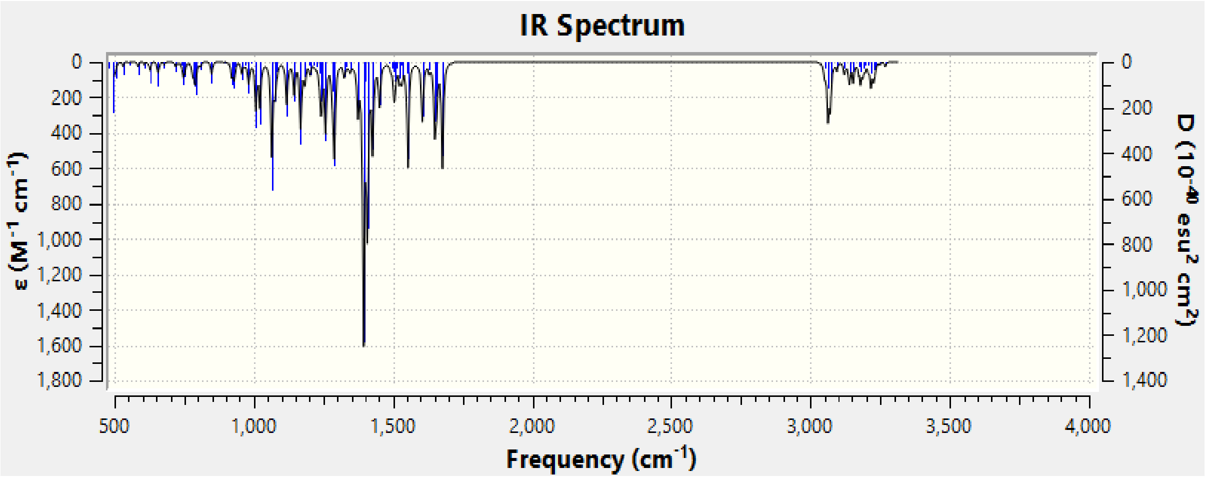
IR Spectrum
ε (M⁻¹ cm⁻¹)
0
200
400
600
800
1,000
1,200
1,400
1,600
1,800
D (10⁻⁴⁰ esu² cm²)
0
200
400
600
800
1,000
1,200
1,400
500
1,000
1,500
2,000
2,500
3,000
3,500
4,000
Frequency (cm⁻¹)

OP10

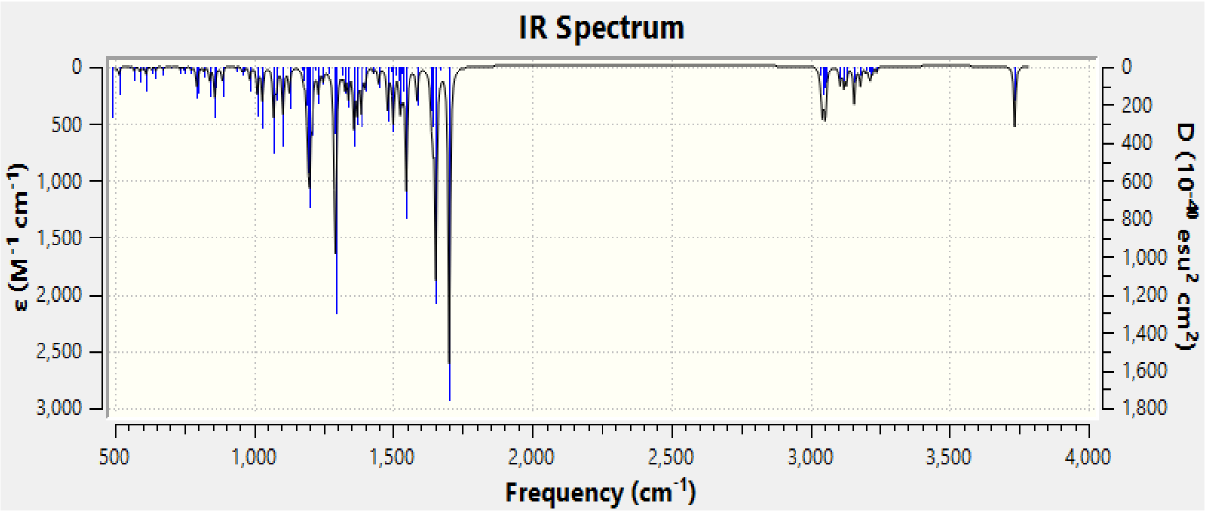

SI Figure 04: Calculated IR spectra of oxidation products by using B3LYP/6-31G(d) basis set and acetonitrile solvent medium.

MALDI-TOF MS studies

(A)

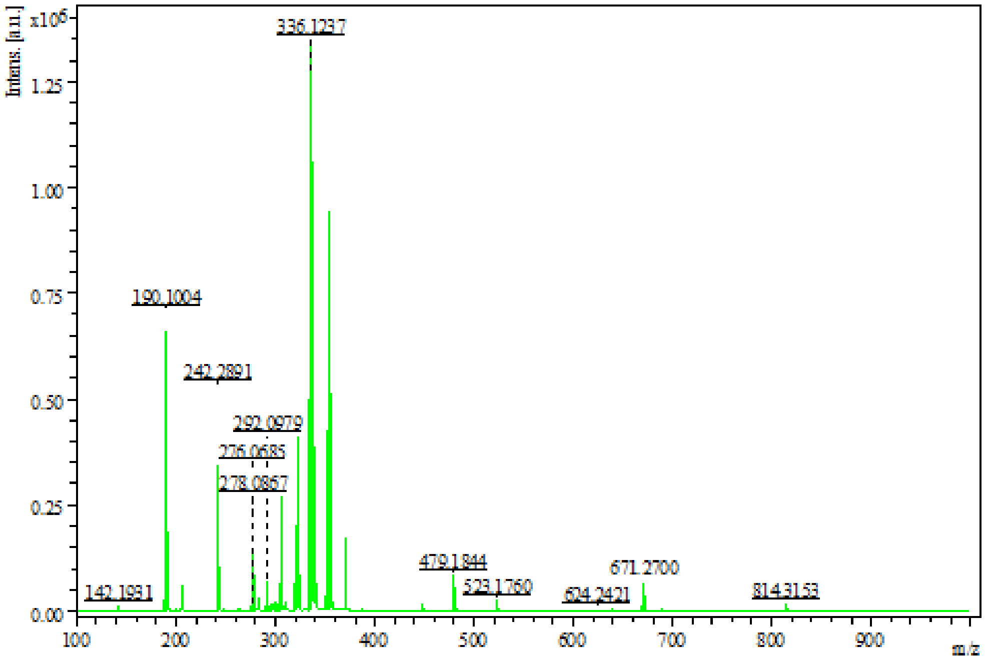

(B)

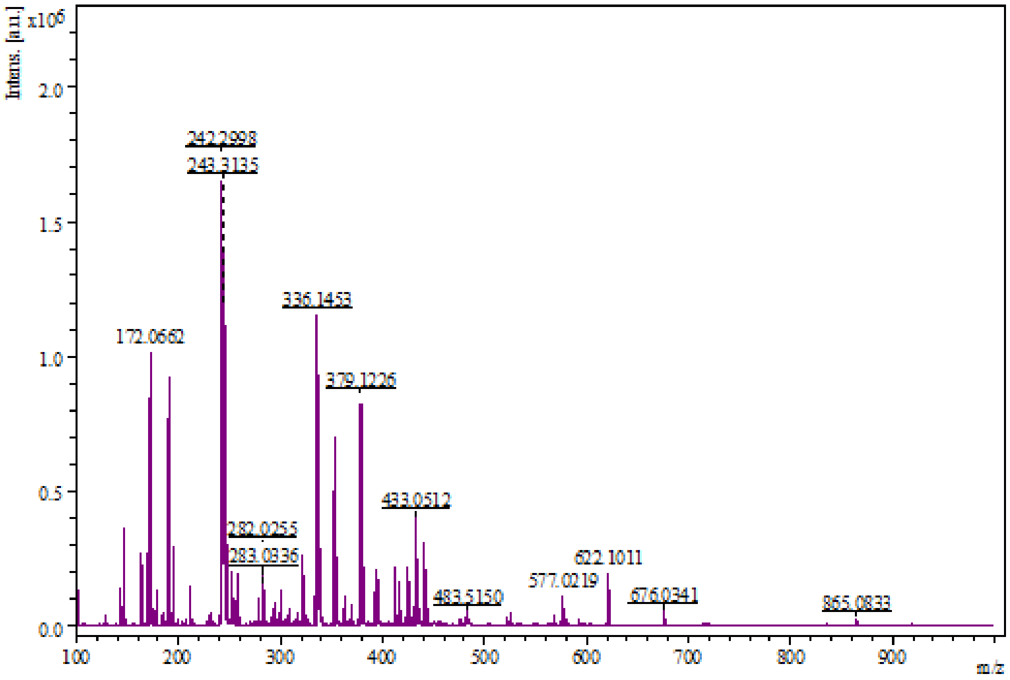

SI Figure 05: (A) MALDI-TOF Mass spectrum (MS) of the Berberine ionized in the matrix (alpha-cyano-4-hydroxycinnamic acid (CHCA)). (B) MALDI-TOF Mass spectrum of the suspected Berberine –EDOT polymer ionized in the matrix matrix (alpha-cyano-4-hydroxycinnamic acid (CHCA))

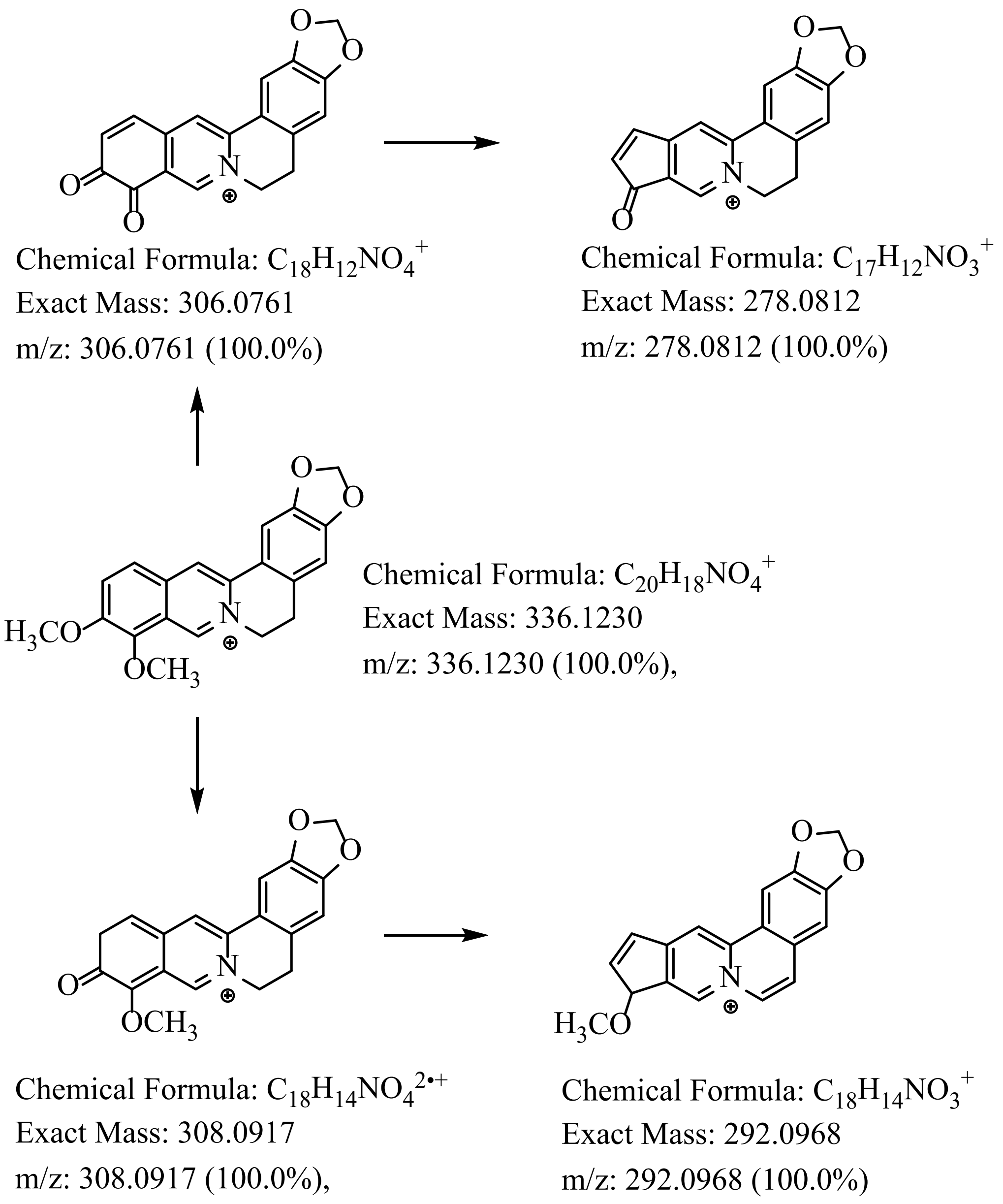

SI Figure 06: Proposed fragmentation pathways of the Berberine

Firstly, the MS fragmentation behavior of Berberine(BBR) was investigated MALTI-TOF MS analysis in positive ion mode (collision energy, 35 eV) (Fig. 9 A). BBR showed [M]+at m/z 336.1234 (elemental composition $C_{20}H_{18}NO_4)^+$. BBR provided abundant fragment ions at m/z 278.0867 (elemental composition $C_{17}H_{12}NO_3)^+$ via the loss of the $2CH_3$ and CO respectively, at m/z 292.0979(elemental composition $C_{18}H_{14}NO_3)^+$ via the loss of the CH_3, H^+ and CO respectively. The proposed fragmentation pathways of BBR were shown in Fig. 10.

Then MS fragmentation behavior of the suspected polymer (Berberine-EDOT) was investigated. Fig 9 B shows the MALDI-TOF mass spectrum for the suspected polymer (Berberine-EDOT). The MALDI has fragments m/z 622.1001, 592.1432 and 579.0340 showing Ber-EDOT+ and PF6- ion pairs. According to the Fig 10 the m/z 622.1001 peak is

strong evidence that shows the polymer has EDOT and Berberine units covalently attached and confirm that it is a copolymer of the two monomers.

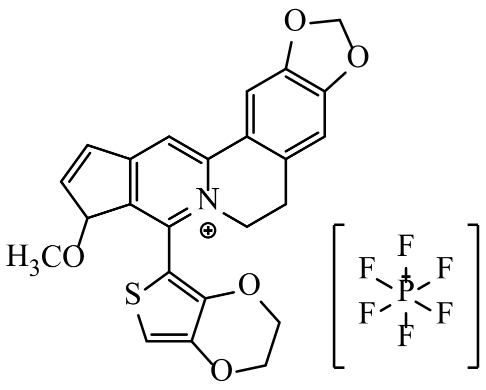

Chemical Formula: $C_{24}H_{20}F_6NO_5PS$
Exact Mass: 579.0704
m/z: 579.0704 (100.0%)

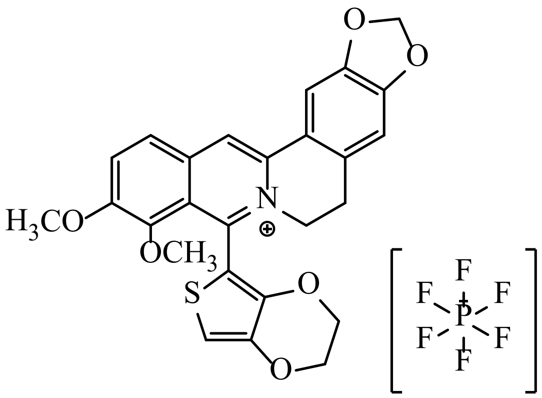

Chemical Formula: $C_{26}H_{22}F_6NO_6PS$
Exact Mass: 621.0810
m/z: 622.0843 (28.1%)

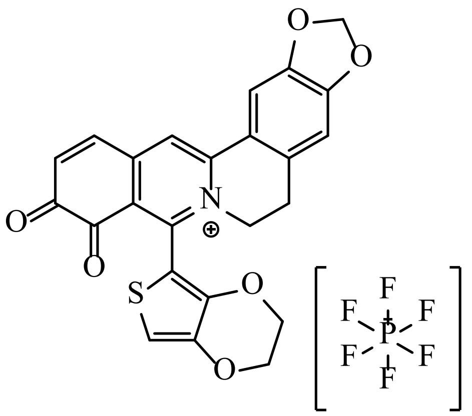

Chemical Formula: $C_{24}H_{16}F_6NO_6PS$
Exact Mass: 591.0340
m/z: 592.0374 (26.0%)

Figure 07: Proposed Berberine – EDOT polymer fragments corresponds to the peaks in the MALDI-TOF mass spectrum.
